# A Physics-Informed Machine Learning Framework for Modeling Biaxial Strain-Induced Band Gap Variation in Zig-Zag Single-Walled Carbon Nanotubes

**DOI:** 10.3390/nano16171114

**Published:** 2026-09-03

**Authors:** Necati Vardar

**Affiliations:** Department of Electrical and Electronics Engineering, Faculty of Engineering and Natural Sciences, KTO Karatay University, Konya 42020, Turkey; necati.vardar@karatay.edu.tr

**Keywords:** zig-zag SWCNTs, biaxial strain, band gap variation, physics-informed machine learning, target reformulation, leave-one-chirality-out validation

## Abstract

Understanding the strain-dependent electronic response of carbon nanotubes is important for nanoelectronic applications in which mechanical deformation serves as a functional tuning mechanism. This study developed a physics-informed machine learning framework for modeling biaxial strain-induced band gap variation in zig-zag single-walled carbon nanotubes (SWCNTs). A traceable density functional theory/Perdew–Burke–Ernzerhof (DFT/PBE) dataset was reconstructed from published band gap curves and comprised 144 records representing 16 zig-zag SWCNT chirality groups, from (8,0) to (23,0), at nine nominal biaxial strain levels between −10% and +10%. The absolute band gap was reformulated as a baseline-relative target ($\Delta E_{g}$) representing the strain-induced variation from the chirality-specific unstrained state; this formulation assumes that the corresponding unstrained band gap is available for each chirality before prediction. Physics-informed descriptors included the chirality-derived diameter, chirality family, nominal strain, squared strain, and family–strain interaction terms. Predefined linear and nonlinear regression models were evaluated using repeated shuffled five-fold cross-validation, while transfer to unseen chirality groups was assessed using leave-one-chirality-out (LOCO) validation. Sensitivity analyses were additionally conducted to evaluate baseline uncertainty and digitization confidence. Within the represented chirality–strain domain, the best nonlinear configuration—support vector regression using diameter, squared strain, and family–strain descriptors—achieved a mean cross-validated coefficient of determination ($R^{2}$) of 0.880 for $\Delta E_{g}$, compared with 0.786 for absolute $E_{g}$. Ridge regression using family–strain descriptors achieved a mean $R^{2}$ of 0.527, demonstrating that the baseline-relative target also contained an interpretable linear response component. The benefit of target reformulation was most evident in explained variance and fold-level stability, whereas differences in absolute error metrics were comparatively small. The best $\Delta E_{g}$ model achieved a mean LOCO $R^{2}$ of 0.636, although substantial fold-level variation indicated that transfer remained strongly dependent on the held-out chirality group. The findings remained stable under chirality-specific $E_{g0}$ perturbations of up to ±0.020 eV and after exclusion of three low-confidence digitized records. Overall, baseline-relative target formulation and chirality-aware descriptor design improved the modeling of strain-induced electronic responses under limited-data conditions. The results should nevertheless be interpreted as a proof of concept within the represented zig-zag SWCNT domain rather than as a universal predictor across the complete CNT design space.

## 1. Introduction

Nanotechnology enables the design and control of matter at dimensions where quantum, surface, and confinement effects can produce properties that differ substantially from those of bulk materials. These characteristics have supported applications in nanoelectronics, energy technologies, sensing, biomedical systems, environmental monitoring, and advanced composites. In parallel, machine learning (ML) has become increasingly important in nanomaterial research for property prediction, materials screening, structure optimization, synthesis planning, characterization, and quality assessment [1,2,3].

Among carbon-based nanostructures, carbon nanotubes (CNTs) remain particularly important because of their quasi-one-dimensional geometry, high aspect ratio, strong covalent (${sp}^{2}$) bonding, mechanical resilience, and tunable electronic behavior. Their properties can be modified through chirality, diameter, defects, functionalization, environmental interactions, and mechanical deformation, supporting continued research across electronic, sensing, composite, biomedical, and energy-related applications [4]. For strain-dependent electronic modeling, CNTs offer a particularly informative one-dimensional platform because circumferential quantum confinement converts the two-dimensional graphene lattice into discrete chirality-dependent electronic states. Unlike planar graphene, whose pristine form is gapless, semiconducting CNTs can exhibit finite band gaps that vary systematically with chirality, diameter, curvature, and mechanical deformation. This combination of one-dimensional confinement and structurally encoded electronic response makes CNTs especially suitable for studying mechanically tunable band-gap behavior [4,5,6].

The electronic behavior of a CNT is strongly governed by its chirality indices $\left(n,m\right)$, which determine the wrapping geometry of the graphene lattice and define structural characteristics such as diameter, chiral angle, and symmetry. These parameters also influence whether a CNT exhibits metallic-like, small-gap, or semiconducting behavior. The $\left(n-m\right)\;mod\;3$ classification provides a useful first-order description of the CNT electronic family, although curvature, deformation, many-body interactions, and computational methodology may modify the idealized electronic structure [4,5,6]. Because the band gap ($E_{g}$) directly influences carrier transport, switching behavior, optical response, and device sensitivity, understanding its response to structural and mechanical perturbations remains a central problem in CNT-based electronics.

Controllable band gap modulation is relevant to several CNT application areas. In nanoelectronic systems, changes in $E_{g}$ influence the suitability of CNTs for transistor channels, switching elements, and related device components. In flexible and wearable sensing systems, CNT networks and CNT-based composites can convert mechanical deformation into measurable electrical changes, supporting applications in motion monitoring, human–machine interfaces, soft robotics, and mechanically responsive devices [7,8]. CNTs have also been investigated in biosensors, therapeutic-delivery platforms, bioimaging systems, and flexible biomedical interfaces, although such applications require careful evaluation of biocompatibility and safety [9,10,11]. These application areas collectively motivate a clearer understanding of the relationship between CNT structure, mechanical deformation, and electronic response.

Mechanical strain provides a particularly useful route for tuning CNT electronic properties because it can alter the band structure without necessarily introducing chemical dopants or permanent defects. Axial, radial, torsional, and biaxial deformation can modify C–C bond lengths, bond angles, lattice symmetry, and π-orbital overlap. Consequently, strain may increase or decrease the band gap depending on chirality, strain direction, deformation mode, and magnitude. Theoretical studies have demonstrated strong chirality- and deformation-dependent electronic responses [12], while experimental evidence has confirmed that CNT band gaps can be mechanically modulated [13]. First-principles investigations have also reported reversible band gap engineering and deformation-induced electronic transitions [14]. More specifically, DFT/PBE calculations for biaxially strained zig-zag single-walled carbon nanotubes (SWCNTs) have shown substantial differences in band gap response across chirality groups, supporting biaxial strain as a possible design variable for tunable nanoelectronic systems [15].

Systematic investigation of these relationships remains challenging. Experimental studies require CNTs with known chirality, controlled deformation, and reliable electronic characterization, while measurements may also be affected by substrate interactions, electrical contacts, and environmental conditions. Density functional theory (DFT) provides detailed atomistic and electronic information but is computationally demanding when broad combinations of chirality and strain are considered. Available strain-dependent CNT data are therefore often limited to selected structures, deformation conditions, and computational protocols.

Data-driven modeling offers a complementary strategy for extracting structure–property relationships from limited computational data and reducing the need for exhaustive first-principles exploration. ML has been applied broadly in materials science and nanotechnology for property prediction, materials screening, and structure–property learning [1,2,3,16,17,18]. Recent CNT-focused studies have demonstrated ML-based prediction of intrinsic electronic properties such as band gaps [19], while ML-based approaches have also been reported for predicting the electronic structure of quasi-one-dimensional materials under strain [20]. However, these studies typically address either intrinsic electronic-property prediction or deformation-dependent electronic responses through model formulations that differ substantially from the present low-data, chirality-grouped band-gap problem. In particular, comparatively limited attention has been given to whether the strain-induced band-gap response itself can be reformulated relative to a chirality-specific unstrained reference and learned using compact physically motivated descriptors under explicit unseen-chirality validation. Successful low-data modeling therefore depends not only on algorithm choice but also on how the target variable and descriptor space are physically formulated.

This issue is especially important in CNT band gap modeling. Absolute $E_{g}$ values reflect both the intrinsic electronic structure associated with each chirality and the change produced by mechanical deformation. When multiple chirality groups are combined, a model trained directly on absolute $E_{g}$ must learn these baseline differences and the strain-induced response simultaneously. Under limited-data conditions, this combined variation can obscure the deformation-dependent signal, particularly because CNT band gap responses may be nonlinear and asymmetric under tensile and compressive strain.

To address this problem, the present study evaluates a physics-informed target reformulation. The target was defined as the strain-induced deviation from the chirality-specific unstrained state, $\Delta E_{g}=E_{g}-E_{g0}$, where $E_{g0}$ denotes the unstrained band gap of the corresponding chirality. By treating mechanical strain as a perturbation relative to this intrinsic electronic reference state, the formulation was intended to reduce baseline-driven target heterogeneity and focus the learning task more directly on the electromechanical response of interest.

The primary dataset comprised a balanced set of biaxially strained zig-zag SWCNTs reconstructed from the internally consistent DFT/PBE curves reported by Mou et al. [15].

The modeling framework compared absolute and baseline-relative targets using compact physics-informed descriptor sets incorporating the diameter, chirality family, nominal biaxial strain, squared strain, and family–strain interactions. Here, “physics-informed” refers to physically motivated target and descriptor construction rather than to an explicit governing-equation constraint. Interpretable linear and nonlinear models were evaluated using repeated shuffled five-fold cross-validation and leave-one-chirality-out validation, with additional sensitivity analyses addressing baseline and digitization uncertainty.

The principal contribution of this study is the formulation and evaluation of a low-data modeling strategy for the strain-induced CNT electronic response rather than the introduction of a new machine-learning algorithm. Specifically, the study combines a chirality-specific baseline-relative target, compact physically motivated structural and family-dependent descriptors, repeated within-domain validation, and explicit leave-one-chirality-out testing within a traceable single-source DFT/PBE dataset. This framework provides a controlled means of separating intrinsic chirality-dependent electronic baselines from the deformation-induced response and of distinguishing interpolation from transfer to unseen nanotube structures. More broadly, the workflow illustrates how physically motivated target construction and group-aware validation can be incorporated into future low-data modeling studies of strain-tunable nanomaterials. The present results are therefore interpreted as a proof of concept within the represented zig-zag SWCNT domain rather than as a universal predictor across the complete CNT design space.

## 2. Materials and Methods

### 2.1. Data Reconstruction and Dataset Construction

The primary regression dataset was reconstructed from a single internally consistent DFT/PBE source reporting biaxial strain–band gap relationships for zig-zag SWCNTs [15]. This single-source strategy reduced methodological heterogeneity associated with exchange–correlation functional, relaxation protocol, strain definition, and computational setup.

The primary strain-dependent data were derived from the published biaxial strain–band gap curves of Mou et al. [15], who reported density functional theory-based calculations for multiple zig-zag SWCNT chirality groups. The underlying first-principles calculations in the source study are based on the density functional theory framework, which is commonly formulated through the Kohn–Sham equations [21]. Specifically, Figure 5a–c of the source study were used to reconstruct band gap values for 16 zig-zag SWCNTs, including the $\left(8,0\right)$ through $\left(23,0\right)$ chirality range represented across the three chirality-family panels. For each chirality group, nine nominal biaxial strain levels were considered, covering compressive and tensile deformation from −10% to +10% at 2.5% intervals. The resulting primary modeling dataset therefore consisted of 144 strain-dependent records, each corresponding to a defined combination of chirality, biaxial strain level, and band gap value.

Numerical values were reconstructed from 600 dpi renderings of Figure 5a–c using a color-cluster- and marker-based digitization workflow. Each panel was calibrated separately against the labeled nominal-strain and band gap axes, and the extracted marker coordinates were converted to numerical strain and $E_{g}$ values. Visual quality control was subsequently performed, and available text-reported anchor values were used to verify the consistency of the reconstructed values. Because the reconstruction relied on rasterized published figures rather than original tabulated numerical data, finite image resolution, marker overlap or local curve congestion, and pixel-to-data coordinate conversion could introduce small extraction uncertainty. Each reconstructed record was therefore assigned a digitization-confidence label, and visually uncertain points were retained but explicitly flagged for the sensitivity analysis described in Section 2.6.

The reconstructed data were harmonized into a common structure including chirality indices $\left(n,m\right)$, chirality family, nominal biaxial strain percentage, digitized band gap value $E_{g}$, unstrained baseline band gap $E_{g0}$, and source-panel identifier.

Chirality-specific unstrained baselines and the corresponding strain-induced target were derived from the 0% strain records as defined formally in Section 2.3.

After reconstruction, the dataset was checked for duplicate entries, missing values, inconsistent chirality labels, and incomplete strain–band gap records. All 16 chirality groups contained nine nominal biaxial strain levels, producing a balanced 144-record dataset for the primary analysis. No synthetic or interpolation-based records were introduced into the primary modeling dataset. Records with lower digitization confidence were retained in the main dataset but explicitly flagged, and their influence was evaluated through a separate low-confidence point exclusion sensitivity analysis.

**Figure 1:**
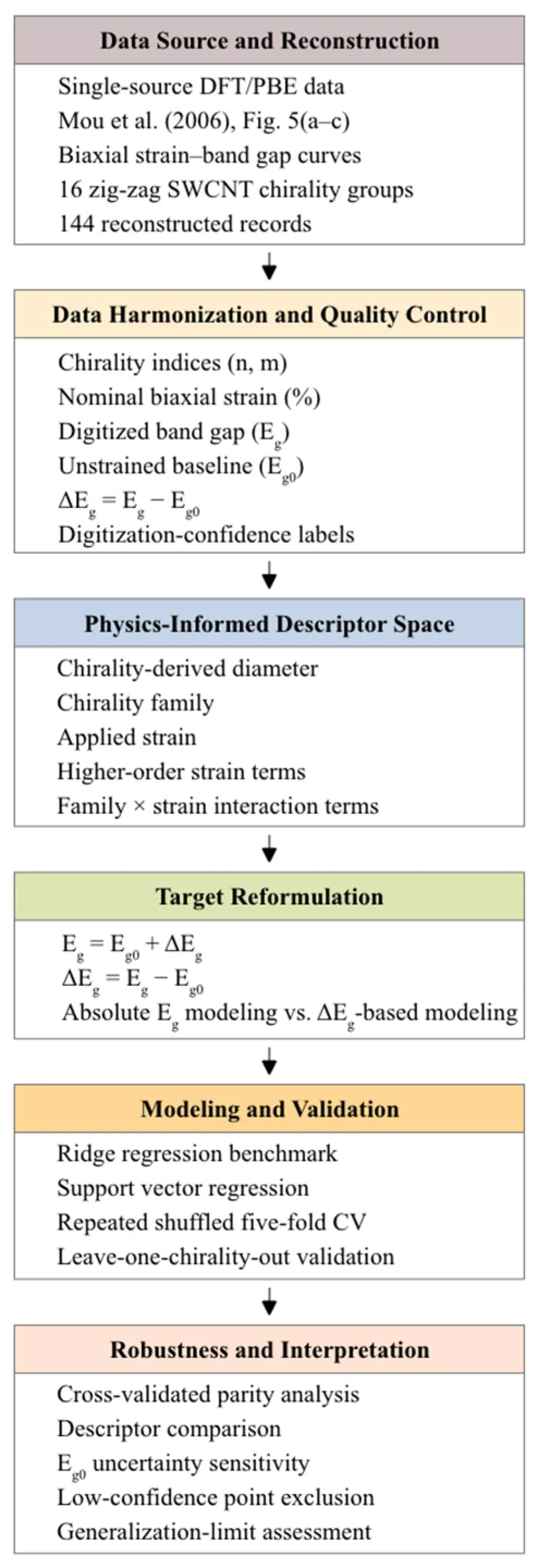
Workflow of the proposed physics-informed machine learning framework for modeling biaxial strain-induced band gap variation in zig-zag SWCNTs. The primary dataset was reconstructed from the single-source DFT/PBE biaxial strain–band gap curves reported by Mou et al. [15], covering 16 zig-zag SWCNT chirality groups and 144 reconstructed records. The data were harmonized by chirality indices, nominal biaxial strain, digitized band gap, unstrained baseline, and digitization-confidence labels. The $E_{g}$ was reformulated as $\Delta E_{g}=E_{g}-E_{g0}$, and physically derived descriptors were evaluated using ridge regression as an interpretable benchmark and support vector regression as a nonlinear model. Model performance was assessed using CV, LOCO validation, $E_{g0}$ uncertainty sensitivity, low-confidence point exclusion, and cross-validated parity analysis.

The complete reconstructed dataset, including chirality, nominal biaxial strain, digitized $E_{g}$, chirality-specific $E_{g0}$, calculated $\Delta E_{g}$, source-panel information, and digitization-confidence labels for all 144 records, is provided in Supplementary Table S1. The overall workflow of the proposed $\Delta E_{g}$-based machine learning framework is summarized in Figure 1.

### 2.2. Physics-Informed Descriptor Space Definition

The descriptor space was designed to balance physical interpretability with sufficient flexibility to represent chirality-family-dependent and nonlinear biaxial strain responses. Rather than relying only on raw chirality indices and applied strain, the modeling workflow evaluated progressively more informative combinations of structural, electronic-family, and deformation-related descriptors. The main descriptor components and their roles in the modeling framework are summarized in Table 1.

The chirality indices (n,m) define CNT wrapping geometry and determine structural properties such as the nanotube diameter, chiral angle, symmetry class, and first-order electronic character [5,6]. Because all primary modeling records represented zig-zag SWCNTs $\left(n,0\right)$, the index m was constant at zero throughout the dataset and was therefore excluded from the numerical feature matrix. It was retained only as part of the conventional chirality notation. Because the chiral angle was 0° for every modeled zig-zag (n,0) SWCNT, it had zero variance and was therefore excluded from all predictive feature matrices.

**Figure 2:**
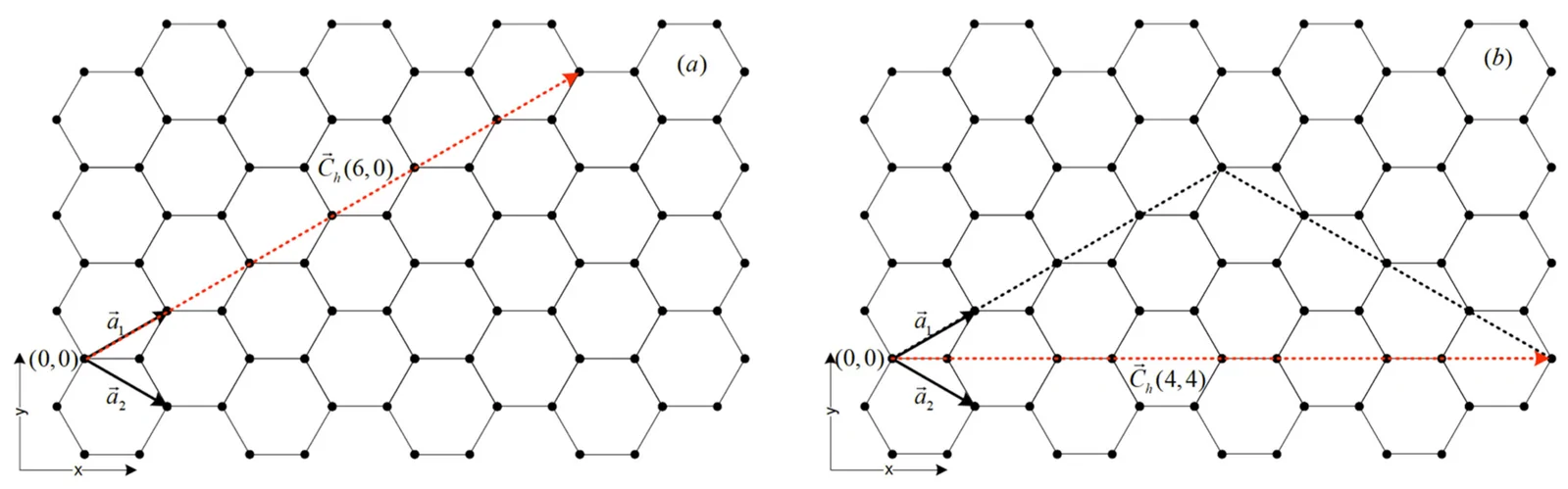
Schematic representation of CNT chirality on a two-dimensional graphene lattice. The chiral vector ${\vec{C}}_{h}=n{\vec{a}}_{1}+m{\vec{a}}_{2}$ defines the wrapping direction of the graphene sheet and determines CNT geometry. Panel (**a**) illustrates a zig-zag configuration $\left(6,0\right)$, whereas panel (**b**) illustrates an armchair configuration $\left(4,4\right)$. The armchair configuration is shown only for structural comparison; the primary predictive dataset consisted exclusively of zig-zag SWCNTs. Schematic illustration adapted from the author’s doctoral dissertation [22]. The red dashed arrow denotes the chiral vector ${\vec{C}}_{h}$, whereas the black dashed lines are auxiliary construction lines illustrating its geometric relation to the graphene lattice vectors.

Table 1 summarizes the physical descriptors used in the modeling workflow. Figure 2 provides complementary structural context by illustrating how the chiral vector defines zig-zag and armchair CNT configurations on a two-dimensional graphene lattice. The armchair configuration shown in Figure 2 was included only to clarify the structural meaning of the chirality indices; armchair and general chiral CNTs were not included in the primary predictive dataset.

The nanotube diameter was calculated from the standard CNT relationship:

(1) $$d=\frac{a}{\pi }\sqrt{n^{2}+nm+m^{2}}$$

where a=2.46 Å is the graphene lattice constant used in the descriptor calculations. For the modeled zig-zag structures, where m=0, the diameter varied primarily with n. Although the diameter is mathematically derived from the chirality indices, it was retained as a physically explicit size descriptor because CNT band gap behavior is influenced by the tube diameter, curvature, and associated changes in electronic structure.

The chirality family was defined using the $\left(n-m\right)\;mod\;3$ classification rule. For zig-zag SWCNTs, this reduces to n mod 3, allowing the modeled nanotubes to be grouped into the 3q, 3q+1, and 3q+2 families. As a first-order electronic classification, the 3q family is nominally metallic-like, whereas the 3q+1 and 3q+2 families are semiconducting-like. This classification was interpreted cautiously because curvature, deformation, and computational methodology may modify the idealized electronic behavior of individual nanotubes. Nevertheless, the chirality family provided a physically meaningful categorical representation of the family-dependent electronic response.

Nominal biaxial strain was used as the primary mechanical descriptor. The strain mode was fixed as biaxial deformation throughout the primary dataset; therefore, the strain mode was not included as a separate varying feature. Negative nominal strain values represented compressive deformation, whereas positive values represented tensile deformation. Because strain-induced band gap responses may be asymmetric, non-monotonic, or nonlinear across compression and tension, a squared strain term was also included in the expanded descriptor formulations.

Family–strain interaction terms were constructed to represent the possibility that the magnitude and direction of the strain response differ among the 3q, 3q+1, and 3q+2 families. These interactions allowed the models to estimate family-dependent strain effects rather than imposing a single global strain relationship across all zig-zag SWCNTs.

The descriptor comparison therefore progressed from minimal representations based on either chirality index n or diameter, together with nominal biaxial strain, to more informative formulations incorporating chirality family, squared strain, and family–strain interaction terms. Because *n* and diameter are perfectly collinear for the modeled zig-zag SWCNTs, they were treated as alternative structural descriptors and were not included simultaneously in the same descriptor set. The most comprehensive descriptor set combined the diameter, nominal biaxial strain, squared strain, chirality-family indicators, and family–strain interaction terms. This feature design remained compact and physically interpretable while allowing nonlinear and family-dependent response components to be evaluated systematically.

### 2.3. Target Variable Reformulation ${(∆E}_{g})$

A central methodological step in this study was the reformulation of the prediction target from absolute $E_{g}$ to the strain-induced band gap variation. This transformation was introduced to reduce the strong baseline variability arising from the chirality-dependent electronic structure and to improve the physical coherence of the learning task. In the modeling workflow, both absolute $E_{g}$ and $\Delta E_{g}$ were evaluated as alternative target formulations in order to directly assess whether modeling the strain-induced deviation improves learnability relative to direct $E_{g}$ prediction.

In CNTs, $E_{g}$ is primarily determined by intrinsic structural parameters, particularly chirality indices (n,m), diameter, and electronic family. As a result, direct prediction of $E_{g}$ requires the model to learn both the intrinsic chirality-dependent baseline and the strain-induced deviation simultaneously. Under limited-data conditions, this combined prediction task can obscure the deformation-specific response and reduce model stability. To address this issue, the band gap was decomposed as:

(2) $$E_{g}=E_{g0}+\Delta E_{g}$$

where $E_{g0}$ represents the unstrained band gap for a given chirality configuration, and $\Delta E_{g}$ represents the deviation induced by applied strain.

For each zig-zag SWCNT chirality group in the reconstructed dataset, $E_{g0}$ was defined as the digitized band gap value at the 0% biaxial strain condition within the same DFT/PBE strain series. This chirality-specific baseline definition ensured that each nanotube was normalized relative to its own unstrained electronic reference state rather than to a global or externally fitted baseline.

The transformed target variable was then calculated as:

(3) $$\Delta E_{g}=E_{g}-E_{g0}$$

where $E_{g}$ denotes the band gap under a given nominal biaxial strain level, and $E_{g0}$ denotes the corresponding unstrained reference value for the same chirality. The $\Delta E_{g}$ formulation therefore assumes that the chirality-specific unstrained reference value is available before prediction; the model estimates the deformation-induced change relative to this known baseline rather than predicting the complete absolute band gap without prior structural reference information. In this sense, $\Delta E_{g}$ is not merely a statistical transformation but a physically meaningful representation of how mechanical deformation perturbs the intrinsic electronic state of a CNT.

Because the reconstructed dataset was derived from published figure-based values, the definition of $E_{g0}$ was also treated as a potential source of baseline uncertainty. To examine whether the modeling conclusions were sensitive to small baseline deviations, an additional chirality-specific $E_{g0}$ perturbation analysis was performed as a robustness check. In this analysis, the unstrained baseline values were independently perturbed at the chirality level, and model performance was re-evaluated to determine whether the $\Delta E_{g}$-based conclusions remained stable under plausible digitization-related baseline uncertainty.

By separating the chirality-dependent baseline from the deformation-induced response, the target transformation was expected to reduce target-space heterogeneity and make the strain-response component more learnable. However, this assumption was not treated as self-evident; it was explicitly tested by comparing absolute $E_{g}$-based modeling with $\Delta E_{g}$-based modeling under the same validation framework. This approach is particularly relevant for low-data nanomaterial modeling, where the number of available chirality–strain combinations is limited and where physically informed target definitions can substantially influence model behavior.

### 2.4. Data Preprocessing and Quality-Control Pipeline

Following the reconstruction and record-level quality-control procedures described in Section 2.1, the complete dataset was converted into model-specific feature–target matrices. Separate matrices were prepared for the absolute band gap $E_{g}$ and strain-induced band gap variation $\Delta E_{g}$ targets using the descriptor combinations defined in Section 2.2.

Descriptor matrices were constructed from the variables defined in Section 2.2. The chirality family was represented using one-hot indicators, and the corresponding family–strain interactions were generated from these indicators and nominal strain. Ridge regularization was used for the encoded family representation, whereas OLS was retained only as an unregularized predictive comparator.

Missing values were assessed across all model-specific feature and target matrices. Records with missing values in essential fields would have been excluded before model fitting; however, the final matrices contained complete values for all variables used in the main analyses.

Feature scaling was applied using z-score standardization for scale-sensitive models:

(4) $$\tilde{x}=\frac{x-\mu }{\sigma }$$

where x denotes the original feature value, μ is the training-fold mean, and σ is the training-fold standard deviation. To prevent information leakage, standardization parameters were estimated exclusively from the training portion of each fold and were then applied to the corresponding validation or test portion. This procedure was used for OLS, ridge regression, and support vector regression. Random forest and gradient boosting regression were evaluated without feature scaling because tree-based splitting is not dependent on the numerical scale of the descriptors.

### 2.5. Model Selection and Comparative Modeling Strategy

Several regression algorithms were evaluated to compare baseline, linear, regularized linear, kernel-based nonlinear, and tree-based ensemble learning strategies. The candidate algorithms included a mean baseline predictor, ordinary linear regression, ridge regression, support vector regression, random forest regression, and gradient boosting regression. The purpose of this comparison was not to identify a universally optimal predictor, but to determine how target formulation, physically informed descriptor design, and model flexibility jointly influenced the learnability of biaxial strain-induced band gap responses.

A mean baseline model was implemented using a training-fold mean predictor and was included to determine whether the candidate regression models learned meaningful structure beyond the average target value. OLS regression was used as an unregularized linear reference, whereas ridge regression was retained as a regularized and interpretable linear benchmark. For ridge regression, the descriptors were standardized within each training fold, and the model was fitted using a regularization parameter of α=1.0 together with an intercept term. The ridge coefficients were estimated by minimizing the following objective function:

(5) $$\underset{w,b}{min}\left\{\sum_{i=1}^{N}{\left[y_{i}-\left(w^{T}{\tilde{x}}_{i}+b\right)\right]}^{2}+\alpha ∥w{∥}_{2}^{2}\right\}$$

where $y_{i}$ denotes the observed target value, ${\tilde{x}}_{i}$ is the standardized descriptor vector for the ith observation, w is the regression coefficient vector, b is the unpenalized intercept term, N is the number of training observations, and α controls the strength of coefficient regularization. In the present analysis, α was fixed at 1.0 and the intercept was fitted using the scikit-learn default setting (fit_intercept=True).

Support vector regression was evaluated using a radial basis function (RBF) kernel to represent nonlinear relationships among the diameter, chirality family, strain magnitude, higher-order strain terms, and family–strain interactions. The support vector regression model was configured using an RBF kernel, C=1.0, epsilon=0.1, and gamma=scale. This model was included because strain-induced CNT band gap responses may be nonlinear, asymmetric between compressive and tensile deformation, and dependent on the chirality family.

Tree-based ensemble models were included as additional nonlinear comparators. Random forest regression was implemented using 300 decision trees, a minimum of three observations per terminal leaf, and a fixed random seed of 42. Gradient boosting regression was implemented using a fixed random seed of 42, while the remaining model settings followed the scikit-learn default configuration. All unspecified parameters for the candidate algorithms were likewise retained at their scikit-learn default values.

Fixed model configurations were used throughout the comparative analysis. No grid search, automated hyperparameter optimization, or nested hyperparameter-tuning procedure was performed. This decision was made to maintain a controlled comparison across target formulations and descriptor sets and to reduce the risk of optimization-driven performance inflation under the present low-data conditions. Accordingly, the reported comparisons should be interpreted as evaluations of predefined model–descriptor configurations rather than as estimates obtained after exhaustive algorithm-specific tuning.

Ridge regression and SVR were emphasized as complementary linear and nonlinear models, while the mean baseline, OLS, random forest, and gradient boosting models served as secondary comparators. All models were evaluated for both absolute and baseline-relative targets using the validation procedures in Section 2.6; representative Ridge and SVR results are reported in the main text, with the complete comparison provided in Supplementary Table S2.

### 2.6. Validation and Generalization Assessment Strategy

Model performance was evaluated using a multi-level validation strategy designed to distinguish within-dataset interpolation from generalization to chirality groups that were entirely absent during model training. The validation framework included CV, LOCO validation, chirality-specific $E_{g0}$ uncertainty analysis, and low-confidence point exclusion. The same validation procedures were applied consistently across the examined target formulations, descriptor sets, and model classes.

Repeated shuffled five-fold CV was used to estimate within-dataset predictive performance. Twenty random seeds (0–19) produced 100 validation folds for each target–descriptor–model configuration, with each record serving once as validation data per repetition. *R*^2^, MAE, and RMSE were calculated for each fold and summarized as mean ± standard deviation across the 100 evaluations.

Because records from the same chirality group could occur in both training and validation subsets, shuffled CV was interpreted as an estimate of within-domain interpolation rather than evidence of transfer to unseen chirality groups.

For the parity analyses, each record received one out-of-fold prediction during each of the 20 repeated cross-validation runs. The resulting 20 out-of-fold predictions per record were averaged to obtain an aggregated prediction for each of the 144 observations. These aggregated predictions were used exclusively for the parity plots, whereas the principal cross-validation results were based on the distribution of the 100-fold-level performance values.

LOCO validation assessed transfer to unseen chirality groups by withholding all nine records of one chirality while training on the remaining 15 groups. Repeating this procedure for all 16 chiralities produced 16 LOCO folds per configuration; $R^{2}$, MAE, and RMSE were calculated per held-out group and summarized across folds.

Negative $R^{2}$ values were retained because they identify held-out chirality groups for which the model did not outperform the corresponding mean-reference prediction.

Additional robustness analyses were performed to evaluate the sensitivity of the reported findings to reconstruction-related uncertainty. Chirality-specific $E_{g0}$ uncertainty was examined by applying independent uniform perturbations separately to the unstrained baseline value of each chirality group. Uncertainty bounds of ±0.005, ±0.010, and ±0.020 eV were evaluated using 500 Monte Carlo realizations per uncertainty level. The perturbation values were generated using a NumPy random number generator with a fixed seed of 123. Each realization was evaluated using the same shuffled five-fold partition generated with a fixed split seed of 42. The same partition was retained across all Monte Carlo realizations so that changes in performance reflected only the imposed $E_{g0}$ perturbations rather than variation arising from different train–validation splits. For each realization, $R^{2}$, MAE, and RMSE were calculated separately for each of the five validation folds and then averaged across the folds to obtain one realization-level value for each metric. The reported uncertainty summaries were subsequently calculated across the 500 realization-level mean values. An unperturbed reference analysis was calculated using the identical validation partition to provide a directly comparable baseline.

The influence of digitization confidence was evaluated through a separate low-confidence point exclusion analysis. The three records classified as low-confidence visual estimates were removed, reducing the dataset from 144 to 141 records. The principal CV and LOCO analyses were then repeated using the reduced dataset. These sensitivity analyses were not used for model selection or hyperparameter tuning; they were conducted solely to determine whether the main findings were materially affected by plausible baseline uncertainty or by the inclusion of visually uncertain digitized records.

### 2.7. Evaluation Metrics and Visualization

Model performance was quantified using the coefficient of determination ($R^{2}$), mean absolute error (MAE), and root mean squared error (RMSE). Multiple complementary metrics were used because $R^{2}$ measures the proportion of variance explained by the model, whereas MAE and RMSE quantify the magnitude of prediction errors in eV. The coefficient of determination was calculated as:

(6) $$R^{2}=1-\frac{\sum_{i=1}^{N}{\left(y_{i}-{\hat{y}}_{i}\right)}^{2}}{\sum_{i=1}^{N}{\left(y_{i}-\bar{y}\right)}^{2}}$$

where $y_{i}$ denotes the observed target value, ${\hat{y}}_{i}$ is the corresponding predicted value, $\bar{y}$ is the mean of the observed target values, and N is the number of observations in the relevant validation subset. Depending on the analysis, the target variable was either the absolute band gap $E_{g}$ or the strain-induced band gap variation Δ$E_{g}$. Negative $R^{2}$ values were retained and interpreted as indicating that the model performed worse than a mean-only reference predictor for the corresponding validation fold or held-out chirality group.

Prediction error was additionally evaluated using MAE and RMSE:

(7) $$\begin{array}{c} MAE=\frac{1}{N}\sum_{i=1}^{N}\left|y_{i}-{\hat{y}}_{i}\right|\\ RMSE=\sqrt{\frac{1}{N}\sum_{i=1}^{N}{\left(y_{i}-{\hat{y}}_{i}\right)}^{2}} \end{array}$$

MAE represents the average absolute prediction error, whereas RMSE gives greater weight to relatively large errors. Both metrics were reported in eV, allowing the magnitude of prediction errors to be interpreted directly in relation to the modeled band gap responses.

Metric summaries followed the fold-level CV and chirality-level LOCO procedures defined in Section 2.6, with minimum and maximum $R^{2}$ additionally retained for LOCO analysis.

In addition to numerical evaluation, prediction behavior was examined using parity plots. The diagonal line represents ideal agreement between observed and predicted values and provides a visual reference for identifying systematic underestimation, overestimation, response-range compression, and dispersion of prediction errors. The parity plots and their displayed metrics were calculated from the aggregated out-of-fold predictions defined in Section 2.6 rather than from full-dataset training fits and may therefore differ slightly from the mean fold-level values reported in Section 3.2.

### 2.8. Computational Environment and Reproducibility

All data preparation, descriptor generation, model development, validation, sensitivity analysis, and visualization procedures were implemented in Python version 3.12.13 using Google Colab as the cloud-based computational environment. Data manipulation and numerical operations were performed using pandas version 2.2.2 and NumPy version 2.0.2 [23,24,25]. Machine-learning models, preprocessing pipelines, and validation procedures were implemented using scikit-learn version 1.6.1 [26], while all figures were generated using Matplotlib version 3.10.0 [27]. Spreadsheet-based validation reports were generated using openpyxl version 3.1.5.

The computational workflow was organized into separate directories for reconstructed input data, processed datasets, model outputs, validation results, robustness analyses, figures, configuration files, and execution logs. The processed modeling dataset, fold-level validation outputs, aggregated out-of-fold predictions, chirality-specific LOCO results, $E_{g0}$-sensitivity outputs, and low-confidence point exclusion results were retained as structured machine-readable files.

Randomized procedures were controlled using explicitly defined seeds. Repeated shuffled five-fold cross-validation used 20 seeds ranging from 0 to 19. Random forest and gradient boosting regression used fixed random seeds of 42. The chirality-specific $E_{g0}$ sensitivity analysis used a fixed five-fold partition generated with a split seed of 42, while independent uniform perturbations were generated using a NumPy random number generator with a fixed seed of 123. For each Monte Carlo realization, $R^{2}$, MAE, and RMSE were calculated separately for each of the five validation folds and then averaged to obtain one realization-level value for each metric.

A configuration file was retained to document the input dataset, target formulations, descriptor sets, model classes, model parameters, validation settings, random seeds, and sensitivity-analysis conditions used in the final workflow. A separate software-version record was generated in JSON format to document the computational environment. The same processed dataset and predefined model configurations were used throughout all comparative analyses. Model fitting and preprocessing were performed through the fold-specific pipelines described in Section 2.5 and Section 2.6, allowing the complete sequence from reconstructed data to reported performance metrics and manuscript figures to be reproduced.

The processed data, analysis code, configuration files, validation outputs, software-version record, spreadsheet-based validation reports, and figure-generation materials are available from the corresponding author upon reasonable request.

## 3. Results

### 3.1. Dataset Characteristics

**Figure 3:**
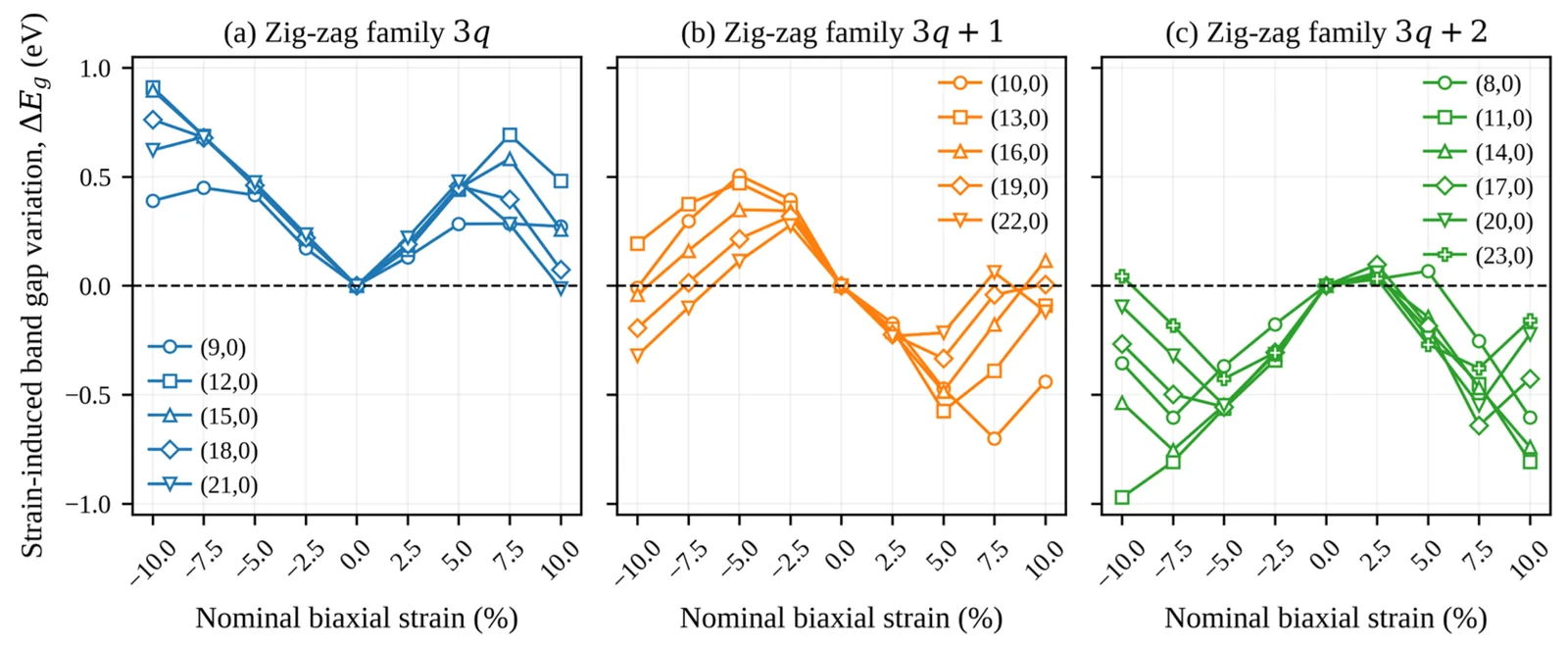
Distribution of reconstructed $∆E_{g}$ values across nominal biaxial strain levels in the primary zig-zag SWCNT dataset. Each point represents one of the 144 reconstructed strain-dependent records obtained from 16 zig-zag chirality groups. The horizontal dashed line indicates $∆E_{g}=0$, corresponding to the chirality-specific unstrained baseline. The spread of positive and negative $∆E_{g}$ values shows that biaxial strain can either increase or decrease the band gap depending on the chirality family and deformation level.

The final dataset comprised 144 records from 16 zig-zag SWCNT chirality groups, with nine biaxial strain levels per group. The baseline-relative target included both positive and negative values, indicating chirality-dependent differences in the direction and magnitude of the strain-induced band-gap response (Figure 3). Dataset characteristics are summarized in Table 2.

Three records were classified as low-confidence digitized points and were retained for the primary analysis while being evaluated separately in the sensitivity analysis.

### 3.2. Repeated Cross-Validation, Target Reformulation, and Descriptor Comparison

Repeated CV compared absolute and baseline-relative targets across the predefined model–descriptor configurations (Table 3; Supplementary Table S2).

For the interpretable linear comparison, ridge regression was evaluated using the same family × strain descriptor set for both target formulations. Direct modeling of absolute $E_{g}$ achieved a mean cross-validated $R^{2}$ of 0.072 ± 0.162, whereas the corresponding Δ$E_{g}$-based model achieved a substantially higher mean $R^{2}$ of 0.527 ± 0.095. These results indicate that removing the chirality-specific unstrained baseline made the deformation-dependent component of the response more accessible to the linear benchmark.

A similar but less pronounced advantage was observed for the nonlinear model. Support vector regression using the diameter + strain^2^ + family × strain descriptor set achieved a mean cross-validated $R^{2}$ of 0.786 ± 0.059 for absolute $E_{g}$, compared with 0.880 ± 0.037 for Δ$E_{g}$. The corresponding MAE and RMSE values were 0.105 and 0.127 eV for absolute $E_{g}$, and 0.101 and 0.128 eV for Δ$E_{g}$, respectively. The advantage of the Δ$E_{g}$-based formulation was therefore most evident in explained variance and fold-level stability, whereas the differences in absolute error metrics were comparatively small.

The results also show that predictive performance depended on descriptor formulation rather than on algorithm choice alone. Minimal chirality–strain representations were insufficient to describe the observed family-dependent and nonlinear strain responses, whereas descriptor sets incorporating the chirality family, diameter, higher-order strain, and family–strain interaction terms produced substantially stronger performance. Ridge regression therefore served as an interpretable benchmark for the linear component of the response, while support vector regression captured additional nonlinear structure.

**Figure 4:**
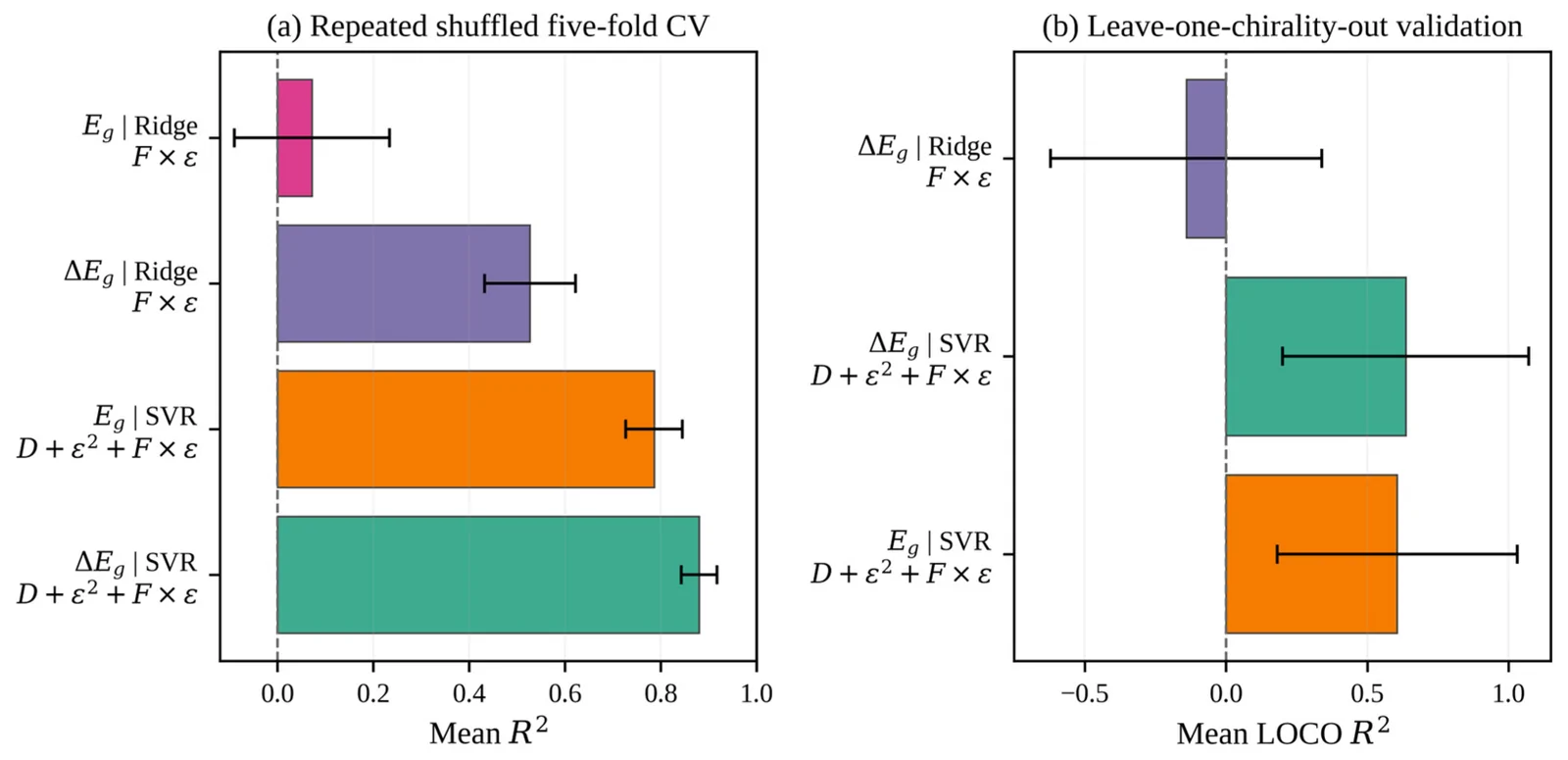
Comparison of representative target–descriptor–model configurations under repeated shuffled five-fold cross-validation and leave-one-chirality-out validation. Panel (**a**) presents mean $R^{2}$ values with standard deviations for the four matched configurations reported in Table 3, calculated across 100 validation folds generated from 20 repetitions of shuffled five-fold cross-validation. Panel (**b**) presents mean LOCO $R^{2}$ values with standard deviations for the three configurations reported in Table 4, calculated across 16 held-out chirality folds. The difference between the panels illustrates the greater difficulty of transferring the learned response to chirality groups that were entirely absent during training. Abbreviations: $E_{g}$, absolute band gap; $\Delta E_{g}$, strain-induced band gap variation; D, diameter; ε, nominal biaxial strain; F, chirality family; SVR, support vector regression; LOCO, leave-one-chirality-out.

Representative repeated-CV results are summarized in Table 3 and Figure 4a, whereas the complete target–descriptor–model comparison is provided in Supplementary Table S2. Across these comparisons, the selected SVR configuration showed the strongest repeated-CV performance.

### 3.3. Chirality-Aware Generalization

LOCO validation evaluated transfer to chirality groups entirely absent from model training and therefore provided a more stringent generalization test than shuffled CV.

The best-performing $\Delta E_{g}$-based model, support vector regression with the diameter + strain^2^ + family × strain descriptor set, achieved a mean LOCO $R^{2}$ of 0.636 ± 0.436, with MAE = 0.105 eV and RMSE = 0.128 eV. The fold-level $R^{2}$ values ranged from −0.879 to 0.938. The positive mean performance indicates that the dataset supports partial transfer to unseen zig-zag chirality groups; however, this transfer was not uniform across individual structures. In particular, the poorest transfer for the principal $\Delta E_{g}$-based SVR configuration occurred when the (9,0) chirality was held out ($R^{2}=-0.879)$, demonstrating that generalization was strongly dependent on the specific chirality excluded from training.

In contrast, the ridge regression benchmark using family × strain descriptors achieved a negative mean LOCO $R^{2}$ of −0.141 ± 0.480. Chirality-specific results further showed pronounced transfer failures for some held-out groups, including (22,0) and (23,0), with the poorest ridge performance observed for (22,0) ($R^{2}=-1.138$). This finding indicates that the interpretable linear model captured a meaningful within-dataset trend but did not transfer reliably to entirely unseen chirality groups. Nonlinear descriptor interactions were therefore important not only for repeated cross-validation performance but also for partial chirality-level generalization. For absolute $E_{g}$, the corresponding support vector regression configuration achieved a mean LOCO $R^{2}$ of 0.606 ± 0.425, with MAE = 0.117 eV and RMSE = 0.133 eV. Although the $\Delta E_{g}$-based model achieved a slightly higher mean LOCO $R^{2}$, its fold-level performance covered a somewhat wider range, from −0.879 to 0.938, compared with −0.565 to 0.942 for absolute $E_{g}$. Thus, the improvement in mean chirality-level performance did not indicate uniform superiority across all held-out chirality groups, and the $\Delta E_{g}$-based model produced a less favorable worst-case result despite its stronger average performance.

Representative LOCO configurations are summarized in Table 4 and Figure 4b, with chirality-specific results for all 16 held-out groups provided in Supplementary Table S3.

### 3.4. Cross-Validated Parity Analysis

**Figure 5:**
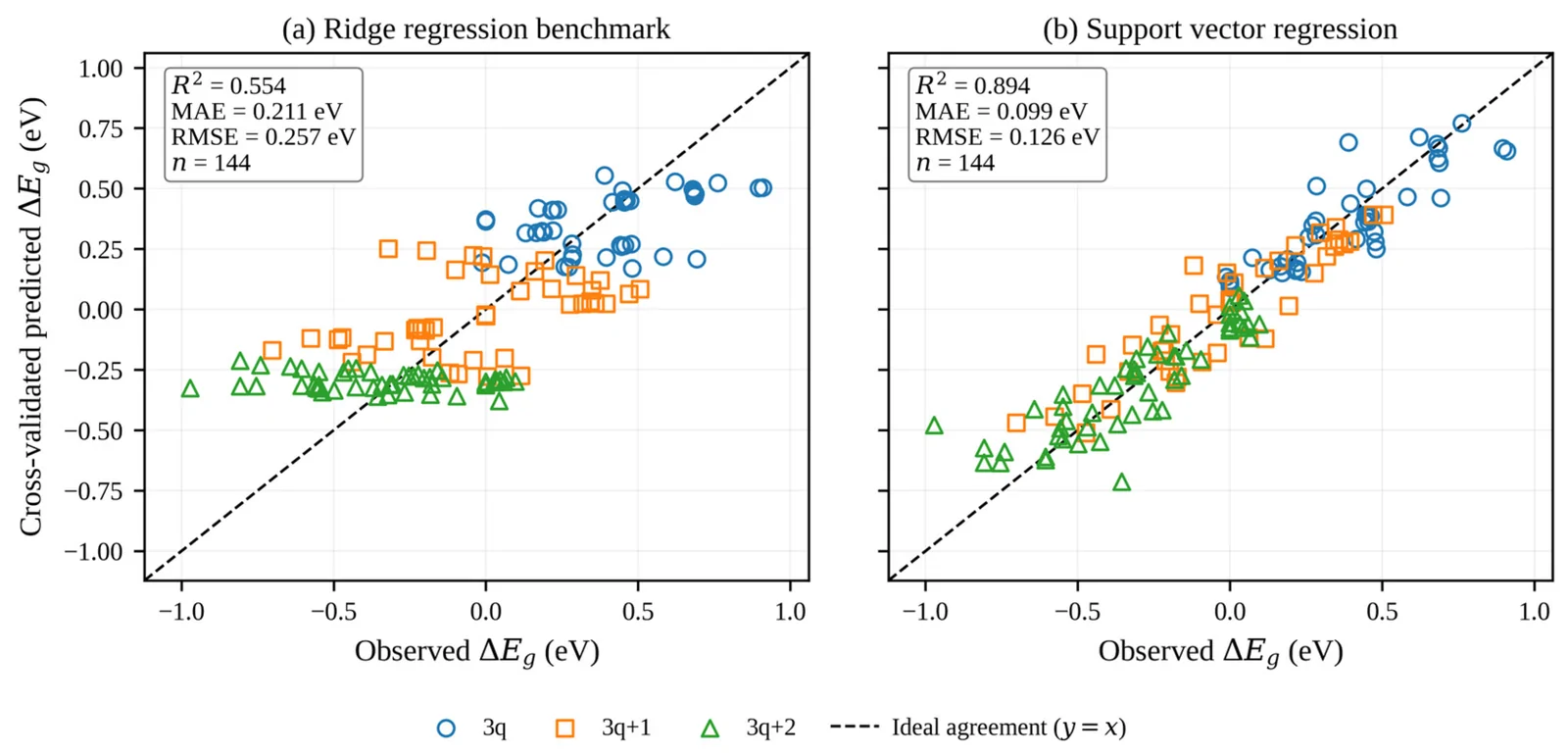
Cross-validated parity plots comparing observed and predicted strain-induced band gap variation $\Delta E_{g}$. Panel (**a**) presents the ridge regression benchmark using family × strain descriptors, whereas panel (**b**) presents support vector regression using the diameter + strain^2^ + family × strain descriptor set. Predictions were generated from aggregated out-of-fold estimates rather than from full-dataset training fits. The dashed diagonal line denotes ideal agreement $\left(y=x\right)$, and symbols distinguish the 3q, 3q+1, and 3q+2 zig-zag chirality families. The inset metrics were calculated from the aggregated out-of-fold predictions for all 144 records and may therefore differ slightly from the mean fold-level cross-validation metrics reported in Table 3.

Cross-validated parity plots were generated from the aggregated out-of-fold predictions defined in Section 2.6 to compare the ridge benchmark with the best-performing SVR configuration (Figure 5).

For the ridge regression benchmark using family × strain descriptors, the aggregated out-of-fold predictions yielded $R^{2}=0.554$, MAE = 0.211 eV, and RMSE = 0.257 eV (Figure 5a). The model captured the overall direction of strain-induced band gap variation, but its predictions were compressed toward the central region of the response distribution. Higher positive $\Delta E_{g}$ values were generally underestimated, whereas strongly negative values were predicted with reduced magnitude. This pattern indicates that the regularized linear benchmark captured a meaningful family- and strain-dependent trend but could not fully represent the nonlinear and high-amplitude responses observed across the complete chirality–strain space.

In contrast, support vector regression using the diameter + strain^2^ + family × strain descriptor set produced substantially closer agreement between observed and predicted $\Delta E_{g}$ values (Figure 5b). The aggregated out-of-fold predictions yielded $R^{2}=0.894$, MAE = 0.099 eV, and RMSE = 0.126 eV. The predictions were distributed more closely around the ideal y=x line and showed less compression at the response extremes than the ridge benchmark. This improvement is consistent with the repeated cross-validation results reported in Table 3 and indicates that nonlinear interactions among the diameter, chirality family, and biaxial strain contributed meaningfully to model performance.

Overall, the parity analysis supports two complementary interpretations. First, the ridge regression benchmark demonstrates that the $\Delta E_{g}$-based formulation contains an interpretable linear strain-response signal, although prediction bias remains at the extremes. Second, the stronger agreement obtained with support vector regression shows that the reconstructed response includes nonlinear and chirality-family-dependent structure that cannot be captured fully by the linear benchmark. Nevertheless, these plots represent within-dataset out-of-fold performance and should not be interpreted as evidence of uniform prediction accuracy for entirely unseen chirality groups, which was evaluated separately through LOCO validation in Section 3.3.

### 3.5. Robustness Analyses

Two complementary robustness analyses were performed to determine whether the main modeling conclusions were sensitive to uncertainty in the chirality-specific unstrained baseline $E_{g0}$ or to the inclusion of visually uncertain digitized records.

First, chirality-specific $E_{g0}$ uncertainty was evaluated by independently perturbing the baseline value of each chirality group within uniform uncertainty ranges of ±0.005, ±0.010, and ±0.020 eV. For each uncertainty level, 500 Monte Carlo realizations were generated and evaluated using the same fixed shuffled five-fold split. An unperturbed reference analysis was also performed under the identical validation protocol to provide a directly comparable baseline. Under the unperturbed reference condition, the $\Delta E_{g}$-based support vector regression model using the diameter + strain^2^ + family × strain descriptor set achieved a mean $R^{2}$ of 0.858, with MAE = 0.107 eV and RMSE = 0.137 eV. The corresponding mean $R^{2}$ values remained 0.858, 0.857, and 0.856 under uncertainty ranges of ±0.005, ±0.010, and ±0.020 eV, respectively. Even under the largest perturbation range, the empirical 2.5th–97.5th percentile interval remained between 0.848 and 0.862. These findings indicate that the predictive performance of the $\Delta E_{g}$-based support vector regression model was not materially affected by small variations in the digitized unstrained baseline values.

Second, the influence of the three records classified as low-confidence digitized points was evaluated by excluding these observations and repeating the principal validation analyses. Their removal reduced the dataset from 144 to 141 records. For the $\Delta E_{g}$-based support vector regression model, CV performance changed from $R^{2}=0.880\pm 0.037$ in the complete dataset to $R^{2}=0.890\pm 0.031$ after exclusion. MAE remained essentially unchanged, whereas RMSE decreased from 0.128 to 0.124 eV. LOCO performance also remained stable, changing from $R^{2}=0.636\pm 0.436$ to $R^{2}=0.659\pm 0.414$, with small reductions in MAE and RMSE.

The robustness results are summarized in Table 5. Overall, the limited changes observed under chirality-specific baseline perturbation and low-confidence point exclusion indicate that the main conclusions were not determined by plausible reconstruction-related uncertainty or by the three visually uncertain records.

## 4. Discussion

### 4.1. Interpretation of the ${\Delta E}_{g}$-Based Target Reformulation

The principal methodological finding of this study is that reformulating the prediction target from the absolute band gap $E_{g}$ to strain-induced band gap variation Δ$E_{g}$ produced a more coherent learning problem for the reconstructed zig-zag SWCNT dataset. Absolute $E_{g}$ reflects both the intrinsic electronic structure of each chirality and the additional change caused by mechanical deformation. Consequently, direct $E_{g}$ prediction requires the model to learn substantial chirality-dependent baseline variation together with the strain-induced response.

By defining $\Delta E_{g}=E_{g}-E_{g0}$, the baseline-relative formulation expressed each strained observation relative to the unstrained reference state of the same chirality. This transformation did not remove the physical differences among CNT structures; rather, it separated the chirality-specific baseline from the deformation-dependent component of interest. The model could therefore focus more directly on how biaxial strain modified the band gap relative to the corresponding unstrained nanotube.

The improved performance observed for the $\Delta E_{g}$-based configurations supports this interpretation. Under matched model and descriptor settings, predicting the strain-induced change was generally more effective than predicting the absolute band gap. The advantage was observed for both the interpretable ridge benchmark and the nonlinear support vector regression model, indicating that the benefit of target reformulation was not restricted to a single algorithmic class. Accordingly, the benefit of target reformulation should be interpreted primarily as improved representation of variance and more stable fold-level performance, rather than as a uniform reduction across all error metrics.

The transformation also improved physical interpretability. Positive $\Delta E_{g}$ values represent band gap opening relative to the unstrained state, whereas negative values represent band gap narrowing. Thus, the target directly describes the electromechanical response induced by biaxial deformation. The result should nevertheless be interpreted within the scope of the present dataset and should not be taken to imply that relative targets will invariably outperform absolute-property prediction in every CNT modeling problem.

### 4.2. Chirality-Family Dependence and Nonlinear Model Behavior

The reconstructed response patterns were consistent with the established understanding that CNT band structures respond to mechanical deformation in a chirality-dependent manner. Previous theoretical and experimental studies have shown that the magnitude and direction of strain-induced band gap changes depend strongly on CNT structure and deformation mode [12,13]. The biaxial strain calculations reported by Mou et al. similarly demonstrated substantial variation among zig-zag SWCNT chirality groups [15].

For the modeled (n,0) nanotubes, the n mod 3 classification provided a physically meaningful first-order representation of the 3q, 3q+1, and 3q+2 electronic families. The reconstructed $\Delta E_{g}$ curves did not follow a common global strain trend. Instead, both the direction and magnitude of the response varied among chirality groups and families. The occurrence of positive and negative $\Delta E_{g}$ values should therefore be understood as evidence of family-dependent electromechanical behavior rather than as random variation around a universal strain response.

This structure explains why descriptor sets containing chirality-family indicators and family–strain interactions performed better than formulations based only on nominal strain or a minimal structural descriptor. A global strain coefficient would implicitly assume a similar response across all modeled nanotubes. Family–strain interaction terms instead allowed the direction and magnitude of the deformation response to differ among the 3q, 3q+1, and 3q+2 classes.

The ridge regression benchmark showed that these physically informed descriptors contained a meaningful linear signal. However, its predictions were compressed toward the central region of the response range, particularly for high-amplitude positive and negative $\Delta E_{g}$ values. This behavior indicates that the regularized linear model captured the general direction of the family-dependent strain response but could not fully represent its nonlinear extremes.

Support vector regression achieved stronger performance when the diameter, strain^2^, chirality family, and family–strain interactions were combined. The improvement is consistent with the presence of nonlinear relationships among nanotube size, strain magnitude, and electronic family. Because the models were evaluated using predefined rather than exhaustively optimized hyperparameters, the result should be interpreted as evidence that nonlinear model flexibility was beneficial under the examined configurations, rather than as a definitive ranking of all possible regression algorithms.

### 4.3. Within-Dataset Prediction and Chirality-Level Transfer

The validation results distinguish two related but different forms of model performance. CV evaluated prediction within the reconstructed chirality–strain domain. Because records from the same chirality group could appear in both training and validation subsets, this procedure primarily measured how effectively the models interpolated among strain-dependent patterns represented within the dataset.

The aggregated out-of-fold parity analysis supported the repeated cross-validation findings. The support vector regression predictions were distributed more closely around the ideal y=x line than those of the ridge benchmark and exhibited less compression at the response extremes. This pattern indicates that the nonlinear configuration captured a substantial portion of the within-dataset variation without relying on full-dataset training-fit predictions.

LOCO validation posed a more stringent question by excluding all nine strain records belonging to one chirality group during each fold. Although the best Δ$E_{g}$-based model retained positive mean LOCO performance, the results varied substantially across the 16 held-out chirality groups. The presence of negative $R^{2}$ values for individual groups indicates that transfer was not uniformly successful and that some chirality-specific response patterns were not adequately represented by relationships learned from the remaining structures. The chirality-specific LOCO results further indicate that these transfer limitations were model-dependent: the poorest $\Delta E_{g}$-based SVR performance occurred for the held-out (9,0) nanotube, whereas the linear ridge benchmark showed pronounced failures for other groups, including (22,0) and (23,0). This heterogeneity suggests that extrapolation becomes more difficult when the strain–band-gap response of a held-out nanotube is insufficiently represented by structurally or electronically similar chirality groups in the training set. The observed variation may reflect the combined influence of the nanotube diameter, curvature-related electronic structure, chirality family, and their strain-dependent response; however, the present dataset does not permit attribution of the LOCO failures to a single microscopic mechanism. Moreover, the slightly higher mean LOCO performance of the Δ$E_{g}$-based SVR model was accompanied by a somewhat wider fold-level range and a lower minimum $R^{2}$ than the corresponding absolute $E_{g}$ model. This finding indicates that improved average transfer performance should not be interpreted as uniformly greater stability or superior prediction for every unseen chirality group.

Repeated cross-validation and LOCO should therefore be interpreted as complementary rather than competing estimates. The former demonstrates that the reconstructed response is learnable when related chirality patterns are represented during training. The latter shows that extrapolation to an entirely unseen chirality remains more difficult and depends on the structural and electronic similarity between the held-out group and those available for model development.

This distinction is particularly important in small materials datasets. High interpolation performance does not necessarily imply reliable prediction for untested structures. Accordingly, the present results support the use of the model within the represented chirality–strain domain while also showing that broader chirality-level generalization requires more diverse training data and independent validation.

### 4.4. Implications for Low-Data Nanomaterial Modeling

The findings illustrate that model performance in low-data materials informatics depends not only on algorithm selection but also on how the prediction problem is formulated. Machine learning has considerable potential to support materials discovery and property prediction [1,2], but its value under constrained data conditions depends on the physical coherence of the target variable, descriptor space, and validation design.

In the present study, the strongest results were obtained by combining three elements: a baseline-relative target, physically informed structural and family descriptors, and a nonlinear model capable of representing interaction effects. Increasing model flexibility alone would not have resolved the heterogeneity caused by pooling chirality-dependent baseline band gaps. Similarly, target reformulation without descriptors representing family-dependent behavior would have provided an incomplete description of the response.

The single-source design strengthened internal methodological consistency but necessarily limited external generalizability, as discussed further in Section 4.5.

The same general principle may be relevant to other materials problems in which an externally induced response is superimposed on a structure-dependent baseline. Examples include strain-induced changes in graphene derivatives, nanoribbons, two-dimensional semiconductors, and functional nanocomposites. In such settings, modeling the change relative to an appropriate reference state may produce a more interpretable response variable than direct prediction of the absolute property.

### 4.5. Limitations and Future Directions

Several limitations define the scope of the present findings. First, the dataset contains 144 records derived from 16 zig-zag SWCNT chirality groups under a single nominal biaxial strain protocol. Armchair CNTs, general chiral CNTs, multi-walled structures, and alternative deformation modes were not represented. The reported performance therefore cannot be generalized directly to the full CNT design space.

Second, the primary dataset was reconstructed from a single DFT/PBE source. This restriction was intentionally adopted to preserve internal consistency in the exchange–correlation functional, structural relaxation protocol, biaxial strain definition, and band gap extraction procedure, thereby avoiding methodological heterogeneity as a confounding source of model variation. However, this internal consistency comes at the cost of external validity: the present models have not been independently validated using datasets generated with different computational protocols, alternative exchange–correlation treatments, higher-level electronic-structure methods, or experimental measurements. Consequently, the reported performance should be interpreted as source- and domain-specific rather than as evidence of transfer across DFT methodologies or the broader CNT design space. Future validation should therefore include independent datasets and alternative electronic-structure treatments, including hybrid-functional or many-body approaches where appropriate, together with experimental benchmarking when comparable strain-dependent data become available.

Third, the numerical records were reconstructed from published graphical curves rather than obtained entirely from original tabulated data. The chirality-specific $E_{g0}$ perturbation analysis and low-confidence point exclusion showed that the main conclusions were stable under the examined uncertainty conditions. Nevertheless, these analyses cannot eliminate all uncertainty associated with figure-based digitization. Future studies should prioritize openly deposited, machine-readable numerical datasets.

Fourth, the $\Delta E_{g}$ formulation assumes that the chirality-specific unstrained reference value is available before prediction. Applications in which the unstrained reference is unavailable would require a separate model for $E_{g0}$ or a joint absolute-property prediction framework.

Fifth, the exclusive use of zig-zag SWCNTs meant that m and the chiral angle were constant and could not be evaluated as independently varying predictors. Broader datasets should include armchair and general chiral structures, wider diameter ranges, multiple strain modes, and more varied computational conditions. Such datasets would permit direct assessment of chiral-angle effects, strain-mode interactions, curvature-related behavior, and transfer among structural families.

Sixth, the model configurations were predefined, and no exhaustive hyperparameter optimization or nested model-selection procedure was performed. This approach reduced the risk of optimization-driven performance inflation and supported a controlled comparison among target–descriptor–model configurations. Although the evaluated model–descriptor configurations were predefined, selecting the strongest-performing configuration from the same repeated cross-validation results may still introduce a degree of model-selection optimism. Independent external validation or nested model-selection procedures will therefore be required to obtain an unbiased estimate of future predictive performance. Larger datasets could additionally support uncertainty-aware kernel methods, Gaussian process regression, graph-based models, and more explicit physics-informed architectures.

Future studies should additionally examine leave-one-family-out and cross-source validation as broader tests of structural and methodological transfer.

Within these boundaries, the present study provides proof-of-concept evidence that baseline-relative target formulation, chirality-aware descriptor construction, and validation at the structure-group level can improve the modeling and interpretation of strain-induced electronic responses in low-data CNT systems.

## 5. Conclusions

This study developed a physics-informed machine-learning framework for modeling biaxial strain-induced band-gap responses in zig-zag SWCNTs using a chirality-specific baseline-relative target.

The $\Delta E_{g}$-based formulation was more learnable than direct prediction of absolute $E_{g}$ under matched model and descriptor conditions. Ridge regression using family × strain descriptors identified an interpretable linear response component, with a mean repeated cross-validated $R^{2}$ of 0.527. The strongest within-dataset performance was obtained using support vector regression with the diameter + strain^2^ + family × strain descriptor set, which achieved a mean repeated cross-validated $R^{2}$ of 0.880. These results indicate that both target reformulation and nonlinear chirality-aware descriptor interactions contributed to predictive performance.

LOCO validation showed that transfer to entirely unseen chirality groups remained more variable than interpolation within the reconstructed dataset. The principal findings nevertheless remained stable under chirality-specific $E_{g0}$ perturbation and low-confidence point exclusion analyses. Accordingly, the proposed framework should be interpreted as a reproducible proof of concept for physics-informed, low-data modeling of strain-induced electronic responses rather than as a universal predictor across the full CNT design space. Broader structural coverage and independent computational or experimental validation will be required to assess generalization beyond the present single-source zig-zag SWCNT dataset.

## Figures and Tables

**Table 1:** Physics-informed descriptors used in the modeling workflow.

Feature or Descriptor	Definition or Calculation	Role in the Present Study
Chirality index n	First index of the CNT chirality notation (n,m)	Retained as a chirality identifier and considered as an alternative structural descriptor; not included simultaneously with diameter because d = (a/π)n for the modeled zig-zag SWCNTs
Chirality index m	Second index of the CNT chirality notation (n,m)	Constant at (m=0); retained in conventional notation but excluded from the numerical feature matrix
Diameter d	$d=(a/\pi )\sqrt{n^{2}+nm+m^{2}}$, with a=2.46 Å	Physically explicit descriptor of nanotube size and curvature-related structural variation; used instead of *n* in the representative model configurations reported in the representative model configurations presented in Section 3.
Chiral angle	Angle between the chiral vector and the zig-zag direction	Constant at 0° for all modeled zig-zag SWCNTs; calculated for completeness but excluded from prediction because it had zero variance
Chirality family	$\left(n-m\right)\;mod\;3$; reduces to (n mod 3) for (n,0) nanotubes	Encodes the (3q), (3q+1), and (3q+2) electronic-family classes
Nominal biaxial strain ε	Applied strain from −10% to +10%	Primary mechanical perturbation; negative values denote compression and positive values denote tension
Squared strain $\varepsilon^{2}$	Square of the nominal biaxial strain value	Represents nonlinear, asymmetric, or non-monotonic strain-response behavior
Family × strain	Interaction between chirality-family indicators and nominal strain	Allows the direction and magnitude of strain response to vary across chirality families

**Table 2:** Summary of the primary modeling dataset used for the ${∆E}_{g}$-based analysis.

Dataset Property	Value/Description
Primary modeling samples	144 strain-dependent records
Material system	Zig-zag single-walled carbon nanotubes (SWCNTs)
Data origin	Single-source DFT/PBE figure-based reconstruction from Mou et al. [15]
Source figure	Figure 5a–c
Chirality groups	16 zig-zag SWCNTs: $\left(8,0\right)$ through $\left(23,0\right)$
Modeled chirality type	Zig-zag CNTs (n,0)
Chirality-family coverage	$\left(3q\right),$ $\left(3q+1\right),$ and $\left(3q+2\right)$ families represented
Strain levels per chirality	9 nominal biaxial strain levels
Strain range	−10% to +10%
Strain increment	2.5%
Response variable(s) examined	$E_{g}$ and $\Delta E_{g}$
Main prediction target	$∆E_{g}$
Baseline reference	Chirality-specific unstrained band gap ($E_{g0}$) at 0% strain
Baseline availability	($E_{g0}$) identified for all 16 chirality groups
Input descriptors	Chirality index n or chirality-derived diameter, chirality family, nominal biaxial strain, squared strain, and family–strain interaction terms; *n* and diameter were not used simultaneously
Band gap unit	eV
Synthetic/interpolated data	None
Digitization-confidence annotation	Included
Low-confidence points	3 records

Note: The primary modeling dataset consisted exclusively of reconstructed biaxial strain-dependent records from a single internally consistent DFT/PBE source. No synthetic or interpolated values were introduced. Chirality-family coverage was defined using the zig-zag n mod 3 classification.

**Table 3:** Repeated shuffled five-fold cross-validation performance under matched target–descriptor–model configurations.

Target Formulation	Descriptor Set	Model	Mean ($R^{2}$)	SD	MAE (eV)	RMSE (eV)	Interpretation
Absolute band gap ($E_{g}$)	Family × strain	Ridge regression	0.072	0.162	0.219	0.267	Absolute-target linear benchmark
Strain-induced variation (Δ$E_{g}$)	Family × strain	Ridge regression	0.527	0.095	0.213	0.257	Physics-informed linear benchmark
Absolute band gap ($E_{g}$)	Diameter + strain^2^ + family × strain	Support vector regression	0.786	0.059	0.105	0.127	Nonlinear absolute-target benchmark
Strain-induced variation (Δ$E_{g}$)	Diameter + strain^2^ + family × strain	Support vector regression	0.880	0.037	0.101	0.128	Best-performing nonlinear configuration

Note: Mean $R^{2}$, MAE, and RMSE values were calculated across 100 validation folds generated from 20 repetitions of shuffled five-fold cross-validation; SD denotes the standard deviation of the fold-level $R^{2}$ values. $E_{g}$ denotes the absolute band gap, whereas $\Delta E_{g}$ denotes the strain-induced band gap variation relative to the chirality-specific unstrained baseline $E_{g0}$. Ridge and support vector regression comparisons were performed using matched descriptor sets across the two target formulations. Complete results for all candidate models and descriptor configurations are provided in Supplementary Table S2. Descriptor-set labels are abbreviated as follows: “family × strain” includes the chirality-family indicators, nominal strain, and family–strain interaction terms, whereas “diameter + strain^2^ + family × strain” additionally includes diameter and squared strain.

**Table 4:** Leave-one-chirality-out validation performance of representative model configurations.

Target	Model	Descriptor Set	Mean ($R^{2}$)	SD	Min ($R^{2}$)	Max ($R^{2}$)	MAE (eV)	RMSE (eV)
Δ$E_{g}$	Ridge regression	Family × strain	−0.141	0.480	−1.138	0.378	0.212	0.252
Δ$E_{g}$	Support vector regression	Diameter + strain^2^ + family × strain	0.636	0.436	−0.879	0.938	0.105	0.128
$E_{g}$	Support vector regression	Diameter + strain^2^ + family × strain	0.606	0.425	−0.565	0.942	0.117	0.133

Note: LOCO validation used chirality as the grouping variable. Each fold withheld all nine strain-dependent records associated with one chirality group, resulting in 16 held-out chirality folds. Negative $R^{2}$ values indicate performance worse than the fold-specific mean reference implied by the $R^{2}$ definition. Complete LOCO model-comparison results are provided in Supplementary Table S2, and chirality-specific LOCO results are reported in Supplementary Table S3. Descriptor-set labels follow the abbreviated definitions provided in the note to Table 3.

**Table 5:** Robustness analyses for $E_{g0}$ uncertainty and low-confidence point exclusion.

Analysis	Model and Validation Condition	$R^{2}$ Summary	MAE (eV)	RMSE (eV)	Interpretation
Unperturbed reference	SVR for Δ$E_{g}$; fixed shuffled five-fold split	0.858	0.107	0.137	Reference under the sensitivity-analysis protocol
$E_{g0}$ perturbation ± 0.005 eV	SVR for Δ$E_{g}$; 500 Monte Carlo realizations	0.858 [0.856−0.859]	0.107	0.137	Stable under small baseline uncertainty
$E_{g0}$ perturbation ± 0.010 eV	SVR for Δ$E_{g}$; 500 Monte Carlo realizations	0.857 [0.853−0.861]	0.107	0.137	Stable under moderate baseline uncertainty
$E_{g0}$ perturbation ± 0.020 eV	SVR for Δ$E_{g}$; 500 Monte Carlo realizations	0.856 [0.848−0.862]	0.108	0.138	Stable under conservative baseline uncertainty
Complete dataset	SVR for Δ$E_{g}$; repeated shuffled five-fold CV, n=144	0.880±0.037	0.101	0.128	Main repeated-CV reference
Low-confidence points excluded	SVR for Δ$E_{g}$; repeated shuffled five-fold CV, n=141	0.890±0.031	0.101	0.124	No performance degradation
Complete dataset	SVR for Δ$E_{g}$; LOCO, n=144	0.636±0.436	0.105	0.128	Main chirality-aware reference
Low-confidence points excluded	SVR for Δ$E_{g}$; LOCO, n=141	0.659±0.414	0.103	0.124	Chirality-aware results remained stable

Note: Chirality-specific $E_{g0}$ perturbations were sampled independently from uniform distributions bounded by the stated uncertainty levels. Values in square brackets represent empirical 2.5th–97.5th percentile intervals across 500 Monte Carlo realizations. The unperturbed reference was calculated using the same fixed shuffled five-fold split as the perturbation analysis and should therefore be distinguished from the 20-seed repeated cross-validation results. Low-confidence point exclusion removed three visually uncertain records.

## Data Availability

The primary numerical dataset used in this study was reconstructed from the publicly available figure-based biaxial strain–band gap data reported by Mou et al. [15]. The complete reconstructed dataset used for model development and validation is provided in Supplementary Table S1. The complete target–descriptor–model comparison, chirality-specific leave-one-chirality-out validation results, and chirality-specific ($E_{g0}$) uncertainty analysis are provided in Supplementary Tables S2–S4, respectively. Additional machine-readable materials generated during the study, including processed datasets, fold-level validation outputs, aggregated out-of-fold predictions, chirality-specific LOCO outputs, configuration files, software-version records, and figure-generation materials, are available from the corresponding author upon reasonable request.

## Supplementary Materials

The following supporting information can be downloaded at: https://www.mdpi.com/article/10.3390/nano16171114/s1, Supplementary Methods: data reconstruction, descriptor construction, preprocessing, modeling, validation, robustness analyses, and reproducibility controls.; Table S1: Complete reconstructed dataset; Table S2: Complete target–descriptor–model comparison; Table S3: Chirality-specific leave-one-chirality-out results; Table S4: Chirality-specific ($E_{g0}$) uncertainty analysis. Reference [15] is cited in the supplementary materials.

## Abbreviations

The following abbreviations and symbols are used in this manuscript:

CNT	Carbon nanotube
SWCNT	Single-walled carbon nanotube
ML	Machine learning
DFT	Density functional theory
PBE	Perdew–Burke–Ernzerhof
$E_{g}$	Band gap
$E_{g0}$	Chirality-specific unstrained band gap
${∆E}_{g}$	Strain-induced band gap variation
CV	Cross-validation
LOCO	Leave-one-chirality-out
OLS	Ordinary least squares
$R^{2}$	Coefficient of determination
MAE	Mean absolute error
RMSE	Root mean squared error
SVR	Support vector regression
RBF	Radial basis function
