# Supplementary material for: A Physics-Informed Machine Learning Framework for Modeling Biaxial Strain-Induced Band Gap Variation in Zig-Zag Single-Walled Carbon Nanotubes
Source: Nanomaterials (Basel). 2026 Sep 3;16(17):1114. doi: 10.3390/nano16171114 (PMC13567476; doi:10.3390/nano16171114)
Supplement: Supplementary file 1 [file nanomaterials-16-01114-s001.zip › nanomaterials-4529519-supplementary.pdf]

Supplementary Information

A Physics-Informed Machine Learning Framework for Modeling Biaxial Strain-Induced Band Gap Variation in Zig-Zag Single-Walled Carbon Nanotubes

Necati Vardar

Department of Electrical and Electronics Engineering, Faculty of Engineering and Natural Sciences, KTO Karatay University, Konya 42020, Turkey; necati.vardar@karatay.edu.tr

Contents

- Supplementary Methods
- Supplementary Table S1. Complete reconstructed dataset
- Supplementary Table S2. Complete target–descriptor–model comparison
- Supplementary Table S3. Chirality-specific leave-one-chirality-out results
- Supplementary Table S4. Chirality-specific Eg0 uncertainty analysis
- Supplementary References

Supplementary Methods

S1. Data source, figure reconstruction, and axis calibration

The primary numerical dataset was reconstructed from the biaxial strain–band gap curves for zig-zag single-walled carbon nanotubes reported by Mou et al. [1]. The analysis was restricted to the three chirality-family panels in source Fig. 5(a–c), thereby preserving a single density functional theory/Perdew–Burke–Ernzerhof computational origin and a common biaxial strain definition across all records.

Source panels were rendered at 600 dpi and calibrated independently against the labeled nominal-strain and band-gap axes. Marker locations were identified using a color-cluster- and marker-guided digitization workflow. Pixel coordinates were converted to nominal biaxial strain and band-gap values using panel-specific linear axis transformations. Text-reported anchor values, where available, were used to verify local calibration and the consistency of reconstructed points.

S2. Record construction, completeness, and confidence classification

Each reconstructed record retained a unique record identifier, chirality indices (n, m), conventional chirality notation, chirality family, chirality-derived diameter, nominal biaxial strain, digitized band gap Eg, chirality-specific unstrained band gap E_{g0}, calculated strain-induced variation ΔE_g, source panel, extraction method, and digitization-confidence classification. The final dataset contained 144 records representing 16 zig-zag SWCNT chirality groups and nine nominal biaxial strain levels per chirality from −10% to +10% in 2.5% increments. No synthetic or interpolation-based records were added.

For each chirality, E_{g0} was taken from the corresponding 0% biaxial strain record and ΔE_g was calculated as $E_g - E_{g0}$. All 16 zero-strain records were verified to satisfy $\Delta E_g = 0$. Duplicate identifiers, incomplete chirality–strain combinations, inconsistent labels, and missing modeling variables were checked before model construction.

Three records were designated as low-confidence visual estimates because of marker overlap or edge ambiguity: F5V4_015, (12,0) at +2.5% strain; F5V4_033, (18,0) at +2.5% strain; and F5V4_091, (8,0) at −10% strain. These records were retained in the primary dataset and explicitly flagged in Supplementary Table S1. Their influence was assessed by repeating the principal validation analyses after exclusion of the three records.

S3. Descriptor construction and categorical encoding

The candidate feature sets were defined as follows: (i) n + strain; (ii) diameter + strain; (iii) three chirality-family indicators + strain; (iv) three chirality-family indicators + strain + family×strain interactions; (v) diameter + three family indicators + strain + family×strain interactions; (vi) n + strain + strain² + n×strain; and (vii) diameter + strain + strain² + three family indicators + family×strain interactions. For the modeled zig-zag structures, m and chiral angle had zero variance and were excluded from predictive feature matrices. Because n and diameter were exactly collinear for zig-zag

$(n, 0)$ SWCNTs, they were treated as alternative structural descriptors and were not included simultaneously.

Chirality family was encoded using three one-hot indicators for the $3q$, $3q + 1$, and $3q + 2$ classes. Family×strain interaction terms were formed by multiplying each indicator by nominal biaxial strain. The full indicator set was retained. Individual OLS family coefficients were not interpreted because the complete indicator set is exactly linearly dependent in the presence of an intercept; ridge regression stabilized estimation through L_2 regularization.

S4. Model configurations and preprocessing

The candidate algorithms were a training-fold mean baseline, ordinary least-squares regression, ridge regression, radial-basis-function support vector regression, random forest regression, and gradient boosting regression. Exact model settings are reported for every target–descriptor–model combination in Supplementary Table S2. No grid search, automated hyperparameter optimization, or nested tuning was performed.

OLS, ridge regression, and support vector regression were evaluated in pipelines containing z-score standardization. For every validation fold, scaling parameters were estimated exclusively from the training partition and applied to the held-out partition. Random forest and gradient boosting models were evaluated without scaling. This fold-specific implementation prevented preprocessing leakage.

S5. Repeated shuffled five-fold cross-validation and aggregated out-of-fold predictions

Within-dataset predictive performance was assessed using shuffled five-fold cross-validation repeated with random seeds 0–19. Each repetition generated five validation folds, producing 100-fold-level evaluations for every target–descriptor–model configuration. R^2, MAE, and RMSE were calculated separately for each fold; Supplementary Table S2 reports the mean values and the standard deviation of fold-level R^2.

For parity analysis, each record received one out-of-fold prediction in each of the 20 repetitions. The 20 predictions for a record were averaged to obtain one aggregated out-of-fold prediction. These aggregated predictions were used only for parity plots and their displayed metrics; principal repeated-cross-validation results were summarized from the 100-fold-level evaluations.

S6. Leave-one-chirality-out validation

Chirality-level transfer was assessed using leave-one-chirality-out validation. In each fold, all nine records belonging to one chirality were withheld, and the model was fitted using the remaining 15 chirality groups. The process was repeated for all 16 groups. R^2, MAE, and RMSE were calculated independently for each held-out chirality. Negative R^2 values were retained because they indicate performance below the fold-specific mean reference implied by the coefficient-of-determination definition. Supplementary Table S3 presents the per-chirality results for the three representative configurations reported in Table 4 of the main manuscript.

S7. Chirality-specific Eg0 uncertainty analysis

Baseline uncertainty was evaluated for the ΔE_g support vector regression configuration using diameter, nominal strain, strain², family indicators, and family×strain interactions. Independent chirality-specific offsets were sampled from uniform distributions bounded by ±0.005, ±0.010, or ±0.020 eV. Five hundred Monte Carlo realizations were generated for each uncertainty level using a NumPy random-number generator with seed 123.

All realizations used the same shuffled five-fold partition generated with split seed 42. Within each realization, R^2, MAE, and RMSE were calculated separately for the five validation folds and averaged to obtain one realization-level value per metric. Summary statistics were then calculated across the 500 realization-level means. The unperturbed reference used the identical fixed partition.

Supplementary Table S4 reports the mean, standard deviation, median, empirical 2.5th–97.5th percentile interval, MAE, and RMSE.

S8. Low-confidence record exclusion

Exclusion of the three low-confidence records reduced the dataset from 144 to 141 records. For the principal ΔE_g support vector regression configuration, repeated-cross-validation R^2 changed from 0.8800 ± 0.0369 to 0.8899 ± 0.0311; RMSE changed from 0.1284 to 0.1244 eV. Mean LOCO R^2 changed from 0.6361 ± 0.4362 to 0.6588 ± 0.4144, while LOCO RMSE changed from 0.1277 to 0.1244 eV. The analysis was used only as a sensitivity check and not for model selection or tuning.

S9. Computational environment and reproducibility controls

Analyses were performed in Python 3.12.13 in Google Colab using pandas 2.2.2, NumPy 2.0.2, scikit-learn 1.6.1, Matplotlib 3.10.0, and openpyxl 3.1.5. Repeated cross-validation used seeds 0–19; random forest and gradient boosting used random_state=42; the fixed partition for E_{g0} uncertainty used split seed 42; and Monte Carlo offsets used random seed 123. Processed datasets, fold-level outputs, aggregated out-of-fold predictions, chirality-specific LOCO outputs, configuration files, software-version records, and figure-generation materials were retained as machine-readable files.

Supplementary Table S1. Complete reconstructed dataset

The table contains all 144 reconstructed records used in the primary analysis. Diameter was calculated from the standard zig-zag SWCNT relationship. “Regular” denotes a reconstructed point not assigned to the low-confidence visual-estimate category. The extraction workflow is described in Supplementary Methods S1–S2.

Record ID	n	m	Chirality	Family	Diameter (Å)	Nominal strain (%)	Digitized E_g (eV)	E_{g0} (eV)	ΔE_g (eV)	Source	Figure/panel	Extraction method	Digitization confidence	Low-confidence flag
F5V4_091	8	0	(8,0)	3q+2	6.2643	-10	0.2500	0.6050	-0.3550	Mou et al. (2026)	Fig. 5(c)	600-dpi marker digitization	Low visual estimate	Yes
F5V4_092	8	0	(8,0)	3q+2	6.2643	-7.5	0.0000	0.6050	-0.6050	Mou et al. (2026)	Fig. 5(c)	600-dpi marker digitization	Regular	No
F5V4_093	8	0	(8,0)	3q+2	6.2643	-5	0.2360	0.6050	-0.3690	Mou et al. (2026)	Fig. 5(c)	600-dpi marker digitization	Regular	No
F5V4_094	8	0	(8,0)	3q+2	6.2643	-2.5	0.4280	0.6050	-0.1770	Mou et al. (2026)	Fig. 5(c)	600-dpi marker digitization	Regular	No
F5V4_095	8	0	(8,0)	3q+2	6.2643	0	0.6050	0.6050	0.0000	Mou et al. (2026)	Fig. 5(c)	600-dpi marker digitization	Regular	No
F5V4_096	8	0	(8,0)	3q+2	6.2643	2.5	0.6350	0.6050	0.0300	Mou et al. (2026)	Fig. 5(c)	600-dpi marker digitization	Regular	No
F5V4_097	8	0	(8,0)	3q+2	6.2643	5	0.6720	0.6050	0.0670	Mou et al. (2026)	Fig. 5(c)	600-dpi marker digitization	Regular	No
F5V4_098	8	0	(8,0)	3q+2	6.2643	7.5	0.3520	0.6050	-0.2530	Mou et al. (2026)	Fig. 5(c)	600-dpi marker digitization	Regular	No
F5V4_099	8	0	(8,0)	3q+2	6.2643	10	0.0000	0.6050	-0.6050	Mou et al. (2026)	Fig. 5(c)	600-dpi marker digitization	Regular	No
F5V4_001	9	0	(9,0)	3q	7.0474	-10	0.5500	0.1600	0.3900	Mou et al. (2026)	Fig. 5(a)	600-dpi marker digitization	Medium	No
F5V4_002	9	0	(9,0)	3q	7.0474	-7.5	0.6090	0.1600	0.4490	Mou et al. (2026)	Fig. 5(a)	600-dpi marker digitization	Medium	No
F5V4_003	9	0	(9,0)	3q	7.0474	-5	0.5760	0.1600	0.4160	Mou et al. (2026)	Fig. 5(a)	600-dpi marker digitization	Medium	No
F5V4_004	9	0	(9,0)	3q	7.0474	-2.5	0.3320	0.1600	0.1720	Mou et al. (2026)	Fig. 5(a)	600-dpi marker digitization	Medium	No
F5V4_005	9	0	(9,0)	3q	7.0474	0	0.1600	0.1600	0.0000	Mou et al. (2026)	Fig. 5(a)	600-dpi marker digitization	Medium	No
F5V4_006	9	0	(9,0)	3q	7.0474	2.5	0.2910	0.1600	0.1310	Mou et al. (2026)	Fig. 5(a)	600-dpi marker digitization	Medium	No
F5V4_007	9	0	(9,0)	3q	7.0474	5	0.4430	0.1600	0.2830	Mou et al. (2026)	Fig. 5(a)	600-dpi marker digitization	Medium	No
F5V4_008	9	0	(9,0)	3q	7.0474	7.5	0.4450	0.1600	0.2850	Mou et al. (2026)	Fig. 5(a)	600-dpi marker digitization	Medium	No
F5V4_009	9	0	(9,0)	3q	7.0474	10	0.4320	0.1600	0.2720	Mou et al. (2026)	Fig. 5(a)	600-dpi marker digitization	Medium	No
F5V4_046	10	0	(10,0)	3q+1	7.8304	-10	0.7449	0.7544	-0.0095	Mou et al. (2026)	Fig. 5(b)	600-dpi marker digitization	Medium	No
F5V4_047	10	0	(10,0)	3q+1	7.8304	-7.5	1.0510	0.7544	0.2966	Mou et al. (2026)	Fig. 5(b)	600-dpi marker digitization	Medium	No
F5V4_048	10	0	(10,0)	3q+1	7.8304	-5	1.2612	0.7544	0.5068	Mou et al. (2026)	Fig. 5(b)	600-dpi marker digitization	Medium	No
F5V4_049	10	0	(10,0)	3q+1	7.8304	-2.5	1.1494	0.7544	0.3950	Mou et al. (2026)	Fig. 5(b)	600-dpi marker digitization	Medium	No
F5V4_050	10	0	(10,0)	3q+1	7.8304	0	0.7544	0.7544	0.0000	Mou et al. (2026)	Fig. 5(b)	600-dpi marker digitization	Medium	No
F5V4_051	10	0	(10,0)	3q+1	7.8304	2.5	0.5822	0.7544	-0.1722	Mou et al. (2026)	Fig. 5(b)	600-dpi marker digitization	Medium	No
F5V4_052	10	0	(10,0)	3q+1	7.8304	5	0.2822	0.7544	-0.4722	Mou et al. (2026)	Fig. 5(b)	600-dpi marker digitization	Medium	No
F5V4_053	10	0	(10,0)	3q+1	7.8304	7.5	0.0532	0.7544	-0.7012	Mou et al. (2026)	Fig. 5(b)	600-dpi marker digitization	Medium	No
F5V4_054	10	0	(10,0)	3q+1	7.8304	10	0.3148	0.7544	-0.4396	Mou et al. (2026)	Fig. 5(b)	600-dpi marker digitization	Medium	No
F5V4_100	11	0	(11,0)	3q+2	8.6135	-10	0.0220	0.9930	-0.9710	Mou et al. (2026)	Fig. 5(c)	600-dpi marker	Regular	No

Record ID	n	m	Chirality	Family	Diameter (Å)	Nominal strain (%)	Digitized E_g (eV)	E_{g0} (eV)	ΔE_g (eV)	Source	Figure/panel	Extraction method	Digitization confidence	Low-confidence flag
												digitization		
F5V4_101	11	0	(11,0)	3q+2	8.6135	-7.5	0.1860	0.9930	-0.8070	Mou et al. (2026)	Fig. 5(c)	600-dpi marker digitization	Regular	No
F5V4_102	11	0	(11,0)	3q+2	8.6135	-5	0.4300	0.9930	-0.5630	Mou et al. (2026)	Fig. 5(c)	600-dpi marker digitization	Regular	No
F5V4_103	11	0	(11,0)	3q+2	8.6135	-2.5	0.6520	0.9930	-0.3410	Mou et al. (2026)	Fig. 5(c)	600-dpi marker digitization	Regular	No
F5V4_104	11	0	(11,0)	3q+2	8.6135	0	0.9930	0.9930	0.0000	Mou et al. (2026)	Fig. 5(c)	600-dpi marker digitization	Regular	No
F5V4_105	11	0	(11,0)	3q+2	8.6135	2.5	1.0400	0.9930	0.0470	Mou et al. (2026)	Fig. 5(c)	600-dpi marker digitization	Regular	No
F5V4_106	11	0	(11,0)	3q+2	8.6135	5	0.7900	0.9930	-0.2030	Mou et al. (2026)	Fig. 5(c)	600-dpi marker digitization	Regular	No
F5V4_107	11	0	(11,0)	3q+2	8.6135	7.5	0.5410	0.9930	-0.4520	Mou et al. (2026)	Fig. 5(c)	600-dpi marker digitization	Regular	No
F5V4_108	11	0	(11,0)	3q+2	8.6135	10	0.1860	0.9930	-0.8070	Mou et al. (2026)	Fig. 5(c)	600-dpi marker digitization	Regular	No
F5V4_010	12	0	(12,0)	3q	9.3965	-10	1.0090	0.0990	0.9100	Mou et al. (2026)	Fig. 5(a)	600-dpi marker digitization	Medium	No
F5V4_011	12	0	(12,0)	3q	9.3965	-7.5	0.7870	0.0990	0.6880	Mou et al. (2026)	Fig. 5(a)	600-dpi marker digitization	Medium	No
F5V4_012	12	0	(12,0)	3q	9.3965	-5	0.5540	0.0990	0.4550	Mou et al. (2026)	Fig. 5(a)	600-dpi marker digitization	Medium	No
F5V4_013	12	0	(12,0)	3q	9.3965	-2.5	0.3150	0.0990	0.2160	Mou et al. (2026)	Fig. 5(a)	600-dpi marker digitization	Medium	No
F5V4_014	12	0	(12,0)	3q	9.3965	0	0.0990	0.0990	0.0000	Mou et al. (2026)	Fig. 5(a)	600-dpi marker digitization	Medium	No
F5V4_015	12	0	(12,0)	3q	9.3965	2.5	0.2650	0.0990	0.1660	Mou et al. (2026)	Fig. 5(a)	600-dpi marker digitization	Low visual estimate	Yes
F5V4_016	12	0	(12,0)	3q	9.3965	5	0.5410	0.0990	0.4420	Mou et al. (2026)	Fig. 5(a)	600-dpi marker digitization	Medium	No
F5V4_017	12	0	(12,0)	3q	9.3965	7.5	0.7920	0.0990	0.6930	Mou et al. (2026)	Fig. 5(a)	600-dpi marker digitization	Medium	No
F5V4_018	12	0	(12,0)	3q	9.3965	10	0.5800	0.0990	0.4810	Mou et al. (2026)	Fig. 5(a)	600-dpi marker digitization	Medium	No
F5V4_055	13	0	(13,0)	3q+1	10.1796	-10	0.8020	0.6081	0.1939	Mou et al. (2026)	Fig. 5(b)	600-dpi marker digitization	Medium	No
F5V4_056	13	0	(13,0)	3q+1	10.1796	-7.5	0.9838	0.6081	0.3757	Mou et al. (2026)	Fig. 5(b)	600-dpi marker digitization	Medium	No
F5V4_057	13	0	(13,0)	3q+1	10.1796	-5	1.0790	0.6081	0.4709	Mou et al. (2026)	Fig. 5(b)	600-dpi marker digitization	Medium	No
F5V4_058	13	0	(13,0)	3q+1	10.1796	-2.5	0.9672	0.6081	0.3591	Mou et al. (2026)	Fig. 5(b)	600-dpi marker digitization	Medium	No
F5V4_059	13	0	(13,0)	3q+1	10.1796	0	0.6081	0.6081	0.0000	Mou et al. (2026)	Fig. 5(b)	600-dpi marker digitization	Medium	No
F5V4_060	13	0	(13,0)	3q+1	10.1796	2.5	0.4106	0.6081	-0.1975	Mou et al. (2026)	Fig. 5(b)	600-dpi marker digitization	Medium	No
F5V4_061	13	0	(13,0)	3q+1	10.1796	5	0.0330	0.6081	-0.5751	Mou et al. (2026)	Fig. 5(b)	600-dpi marker digitization	Medium	No
F5V4_062	13	0	(13,0)	3q+1	10.1796	7.5	0.2174	0.6081	-0.3907	Mou et al. (2026)	Fig. 5(b)	600-dpi marker digitization	Medium	No
F5V4_063	13	0	(13,0)	3q+1	10.1796	10	0.5173	0.6081	-0.0908	Mou et al. (2026)	Fig. 5(b)	600-dpi marker digitization	Medium	No
F5V4_109	14	0	(14,0)	3q+2	10.9626	-10	0.2230	0.7600	-0.5370	Mou et al. (2026)	Fig. 5(c)	600-dpi marker digitization	Regular	No
F5V4_110	14	0	(14,0)	3q+2	10.9626	-7.5	0.0060	0.7600	-0.7540	Mou et al. (2026)	Fig. 5(c)	600-dpi marker digitization	Regular	No
F5V4_111	14	0	(14,0)	3q+2	10.9626	-5	0.2110	0.7600	-0.5490	Mou et al. (2026)	Fig. 5(c)	600-dpi marker digitization	Regular	No
F5V4_112	14	0	(14,0)	3q+2	10.9626	-2.5	0.4460	0.7600	-0.3140	Mou et al. (2026)	Fig. 5(c)	600-dpi marker digitization	Regular	No
F5V4_113	14	0	(14,0)	3q+2	10.9626	0	0.7600	0.7600	0.0000	Mou et al. (2026)	Fig. 5(c)	600-dpi marker	Regular	No

Record ID	n	m	Chirality	Family	Diameter (Å)	Nominal strain (%)	Digitized E_g (eV)	E_{g0} (eV)	ΔE_g (eV)	Source	Figure/panel	Extraction method	Digitization confidence	Low-confidence flag
												digitization		
F5V4_114	14	0	(14,0)	3q+2	10.9626	2.5	0.7970	0.7600	0.0370	Mou et al. (2026)	Fig. 5(c)	600-dpi marker digitization	Regular	No
F5V4_115	14	0	(14,0)	3q+2	10.9626	5	0.6170	0.7600	-0.1430	Mou et al. (2026)	Fig. 5(c)	600-dpi marker digitization	Regular	No
F5V4_116	14	0	(14,0)	3q+2	10.9626	7.5	0.2920	0.7600	-0.4680	Mou et al. (2026)	Fig. 5(c)	600-dpi marker digitization	Regular	No
F5V4_117	14	0	(14,0)	3q+2	10.9626	10	0.0200	0.7600	-0.7400	Mou et al. (2026)	Fig. 5(c)	600-dpi marker digitization	Regular	No
F5V4_019	15	0	(15,0)	3q	11.7456	-10	0.9790	0.0820	0.8970	Mou et al. (2026)	Fig. 5(a)	600-dpi marker digitization	Medium	No
F5V4_020	15	0	(15,0)	3q	11.7456	-7.5	0.7680	0.0820	0.6860	Mou et al. (2026)	Fig. 5(a)	600-dpi marker digitization	Medium	No
F5V4_021	15	0	(15,0)	3q	11.7456	-5	0.5370	0.0820	0.4550	Mou et al. (2026)	Fig. 5(a)	600-dpi marker digitization	Medium	No
F5V4_022	15	0	(15,0)	3q	11.7456	-2.5	0.2990	0.0820	0.2170	Mou et al. (2026)	Fig. 5(a)	600-dpi marker digitization	Medium	No
F5V4_023	15	0	(15,0)	3q	11.7456	0	0.0820	0.0820	0.0000	Mou et al. (2026)	Fig. 5(a)	600-dpi marker digitization	Medium	No
F5V4_024	15	0	(15,0)	3q	11.7456	2.5	0.2670	0.0820	0.1850	Mou et al. (2026)	Fig. 5(a)	600-dpi marker digitization	Medium	No
F5V4_025	15	0	(15,0)	3q	11.7456	5	0.5280	0.0820	0.4460	Mou et al. (2026)	Fig. 5(a)	600-dpi marker digitization	Medium	No
F5V4_026	15	0	(15,0)	3q	11.7456	7.5	0.6650	0.0820	0.5830	Mou et al. (2026)	Fig. 5(a)	600-dpi marker digitization	Medium	No
F5V4_027	15	0	(15,0)	3q	11.7456	10	0.3410	0.0820	0.2590	Mou et al. (2026)	Fig. 5(a)	600-dpi marker digitization	Medium	No
F5V4_064	16	0	(16,0)	3q+1	12.5287	-10	0.4531	0.4946	-0.0415	Mou et al. (2026)	Fig. 5(b)	600-dpi marker digitization	Medium	No
F5V4_065	16	0	(16,0)	3q+1	12.5287	-7.5	0.6554	0.4946	0.1608	Mou et al. (2026)	Fig. 5(b)	600-dpi marker digitization	Medium	No
F5V4_066	16	0	(16,0)	3q+1	12.5287	-5	0.8430	0.4946	0.3484	Mou et al. (2026)	Fig. 5(b)	600-dpi marker digitization	Medium	No
F5V4_067	16	0	(16,0)	3q+1	12.5287	-2.5	0.8385	0.4946	0.3439	Mou et al. (2026)	Fig. 5(b)	600-dpi marker digitization	Medium	No
F5V4_068	16	0	(16,0)	3q+1	12.5287	0	0.4946	0.4946	0.0000	Mou et al. (2026)	Fig. 5(b)	600-dpi marker digitization	Medium	No
F5V4_069	16	0	(16,0)	3q+1	12.5287	2.5	0.2835	0.4946	-0.2111	Mou et al. (2026)	Fig. 5(b)	600-dpi marker digitization	Medium	No
F5V4_070	16	0	(16,0)	3q+1	12.5287	5	0.0096	0.4946	-0.4850	Mou et al. (2026)	Fig. 5(b)	600-dpi marker digitization	Medium	No
F5V4_071	16	0	(16,0)	3q+1	12.5287	7.5	0.3177	0.4946	-0.1769	Mou et al. (2026)	Fig. 5(b)	600-dpi marker digitization	Medium	No
F5V4_072	16	0	(16,0)	3q+1	12.5287	10	0.6104	0.4946	0.1158	Mou et al. (2026)	Fig. 5(b)	600-dpi marker digitization	Medium	No
F5V4_118	17	0	(17,0)	3q+2	13.3117	-10	0.3750	0.6420	-0.2670	Mou et al. (2026)	Fig. 5(c)	600-dpi marker digitization	Regular	No
F5V4_119	17	0	(17,0)	3q+2	13.3117	-7.5	0.1440	0.6420	-0.4980	Mou et al. (2026)	Fig. 5(c)	600-dpi marker digitization	Regular	No
F5V4_120	17	0	(17,0)	3q+2	13.3117	-5	0.0860	0.6420	-0.5560	Mou et al. (2026)	Fig. 5(c)	600-dpi marker digitization	Regular	No
F5V4_121	17	0	(17,0)	3q+2	13.3117	-2.5	0.3360	0.6420	-0.3060	Mou et al. (2026)	Fig. 5(c)	600-dpi marker digitization	Regular	No
F5V4_122	17	0	(17,0)	3q+2	13.3117	0	0.6420	0.6420	0.0000	Mou et al. (2026)	Fig. 5(c)	600-dpi marker digitization	Regular	No
F5V4_123	17	0	(17,0)	3q+2	13.3117	2.5	0.7390	0.6420	0.0970	Mou et al. (2026)	Fig. 5(c)	600-dpi marker digitization	Regular	No
F5V4_124	17	0	(17,0)	3q+2	13.3117	5	0.4580	0.6420	-0.1840	Mou et al. (2026)	Fig. 5(c)	600-dpi marker digitization	Regular	No
F5V4_125	17	0	(17,0)	3q+2	13.3117	7.5	0.0000	0.6420	-0.6420	Mou et al. (2026)	Fig. 5(c)	600-dpi marker digitization	Regular	No
F5V4_126	17	0	(17,0)	3q+2	13.3117	10	0.2160	0.6420	-0.4260	Mou et al. (2026)	Fig. 5(c)	600-dpi marker	Regular	No

Record ID	n	m	Chirality	Family	Diameter (Å)	Nominal strain (%)	Digitized E_g (eV)	E_{g0} (eV)	ΔE_g (eV)	Source	Figure/panel	Extraction method	Digitization confidence	Low-confidence flag
												digitization		
F5V4_028	18	0	(18,0)	3q	14.0948	-10	0.8370	0.0750	0.7620	Mou et al. (2026)	Fig. 5(a)	600-dpi marker digitization	Medium	No
F5V4_029	18	0	(18,0)	3q	14.0948	-7.5	0.7550	0.0750	0.6800	Mou et al. (2026)	Fig. 5(a)	600-dpi marker digitization	Medium	No
F5V4_030	18	0	(18,0)	3q	14.0948	-5	0.5370	0.0750	0.4620	Mou et al. (2026)	Fig. 5(a)	600-dpi marker digitization	Medium	No
F5V4_031	18	0	(18,0)	3q	14.0948	-2.5	0.2950	0.0750	0.2200	Mou et al. (2026)	Fig. 5(a)	600-dpi marker digitization	Medium	No
F5V4_032	18	0	(18,0)	3q	14.0948	0	0.0750	0.0750	0.0000	Mou et al. (2026)	Fig. 5(a)	600-dpi marker digitization	Medium	No
F5V4_033	18	0	(18,0)	3q	14.0948	2.5	0.2650	0.0750	0.1900	Mou et al. (2026)	Fig. 5(a)	600-dpi marker digitization	Low visual estimate	Yes
F5V4_034	18	0	(18,0)	3q	14.0948	5	0.5320	0.0750	0.4570	Mou et al. (2026)	Fig. 5(a)	600-dpi marker digitization	Medium	No
F5V4_035	18	0	(18,0)	3q	14.0948	7.5	0.4710	0.0750	0.3960	Mou et al. (2026)	Fig. 5(a)	600-dpi marker digitization	Medium	No
F5V4_036	18	0	(18,0)	3q	14.0948	10	0.1490	0.0750	0.0740	Mou et al. (2026)	Fig. 5(a)	600-dpi marker digitization	Medium	No
F5V4_073	19	0	(19,0)	3q+1	14.8778	-10	0.2207	0.4151	-0.1944	Mou et al. (2026)	Fig. 5(b)	600-dpi marker digitization	Medium	No
F5V4_074	19	0	(19,0)	3q+1	14.8778	-7.5	0.4293	0.4151	0.0142	Mou et al. (2026)	Fig. 5(b)	600-dpi marker digitization	Medium	No
F5V4_075	19	0	(19,0)	3q+1	14.8778	-5	0.6307	0.4151	0.2156	Mou et al. (2026)	Fig. 5(b)	600-dpi marker digitization	Medium	No
F5V4_076	19	0	(19,0)	3q+1	14.8778	-2.5	0.7332	0.4151	0.3181	Mou et al. (2026)	Fig. 5(b)	600-dpi marker digitization	Medium	No
F5V4_077	19	0	(19,0)	3q+1	14.8778	0	0.4151	0.4151	0.0000	Mou et al. (2026)	Fig. 5(b)	600-dpi marker digitization	Medium	No
F5V4_078	19	0	(19,0)	3q+1	14.8778	2.5	0.1911	0.4151	-0.2240	Mou et al. (2026)	Fig. 5(b)	600-dpi marker digitization	Medium	No
F5V4_079	19	0	(19,0)	3q+1	14.8778	5	0.0819	0.4151	-0.3332	Mou et al. (2026)	Fig. 5(b)	600-dpi marker digitization	Medium	No
F5V4_080	19	0	(19,0)	3q+1	14.8778	7.5	0.3737	0.4151	-0.0414	Mou et al. (2026)	Fig. 5(b)	600-dpi marker digitization	Medium	No
F5V4_081	19	0	(19,0)	3q+1	14.8778	10	0.4210	0.4151	0.0059	Mou et al. (2026)	Fig. 5(b)	600-dpi marker digitization	Medium	No
F5V4_127	20	0	(20,0)	3q+2	15.6608	-10	0.4530	0.5480	-0.0950	Mou et al. (2026)	Fig. 5(c)	600-dpi marker digitization	Regular	No
F5V4_128	20	0	(20,0)	3q+2	15.6608	-7.5	0.2270	0.5480	-0.3210	Mou et al. (2026)	Fig. 5(c)	600-dpi marker digitization	Regular	No
F5V4_129	20	0	(20,0)	3q+2	15.6608	-5	0.0000	0.5480	-0.5480	Mou et al. (2026)	Fig. 5(c)	600-dpi marker digitization	Regular	No
F5V4_130	20	0	(20,0)	3q+2	15.6608	-2.5	0.2340	0.5480	-0.3140	Mou et al. (2026)	Fig. 5(c)	600-dpi marker digitization	Regular	No
F5V4_131	20	0	(20,0)	3q+2	15.6608	0	0.5480	0.5480	0.0000	Mou et al. (2026)	Fig. 5(c)	600-dpi marker digitization	Regular	No
F5V4_132	20	0	(20,0)	3q+2	15.6608	2.5	0.6080	0.5480	0.0600	Mou et al. (2026)	Fig. 5(c)	600-dpi marker digitization	Regular	No
F5V4_133	20	0	(20,0)	3q+2	15.6608	5	0.3100	0.5480	-0.2380	Mou et al. (2026)	Fig. 5(c)	600-dpi marker digitization	Regular	No
F5V4_134	20	0	(20,0)	3q+2	15.6608	7.5	0.0000	0.5480	-0.5480	Mou et al. (2026)	Fig. 5(c)	600-dpi marker digitization	Regular	No
F5V4_135	20	0	(20,0)	3q+2	15.6608	10	0.3260	0.5480	-0.2220	Mou et al. (2026)	Fig. 5(c)	600-dpi marker digitization	Regular	No
F5V4_037	21	0	(21,0)	3q	16.4439	-10	0.6570	0.0350	0.6220	Mou et al. (2026)	Fig. 5(a)	600-dpi marker digitization	Medium	No
F5V4_038	21	0	(21,0)	3q	16.4439	-7.5	0.7180	0.0350	0.6830	Mou et al. (2026)	Fig. 5(a)	600-dpi marker digitization	Medium	No
F5V4_039	21	0	(21,0)	3q	16.4439	-5	0.5090	0.0350	0.4740	Mou et al. (2026)	Fig. 5(a)	600-dpi marker digitization	Medium	No
F5V4_040	21	0	(21,0)	3q	16.4439	-2.5	0.2710	0.0350	0.2360	Mou et al. (2026)	Fig. 5(a)	600-dpi marker	Medium	No

Record ID	n	m	Chirality	Family	Diameter (Å)	Nominal strain (%)	Digitized E_g (eV)	E_{g0} (eV)	ΔE_g (eV)	Source	Figure/panel	Extraction method	Digitization confidence	Low-confidence flag
												digitization		
F5V4_041	21	0	(21,0)	3q	16.4439	0	0.0350	0.0350	0.0000	Mou et al. (2026)	Fig. 5(a)	600-dpi marker digitization	Medium	No
F5V4_042	21	0	(21,0)	3q	16.4439	2.5	0.2560	0.0350	0.2210	Mou et al. (2026)	Fig. 5(a)	600-dpi marker digitization	Medium	No
F5V4_043	21	0	(21,0)	3q	16.4439	5	0.5130	0.0350	0.4780	Mou et al. (2026)	Fig. 5(a)	600-dpi marker digitization	Medium	No
F5V4_044	21	0	(21,0)	3q	16.4439	7.5	0.3180	0.0350	0.2830	Mou et al. (2026)	Fig. 5(a)	600-dpi marker digitization	Medium	No
F5V4_045	21	0	(21,0)	3q	16.4439	10	0.0230	0.0350	-0.0120	Mou et al. (2026)	Fig. 5(a)	600-dpi marker digitization	Medium	No
F5V4_082	22	0	(22,0)	3q+1	17.2269	-10	0.0408	0.3610	-0.3202	Mou et al. (2026)	Fig. 5(b)	600-dpi marker digitization	Medium	No
F5V4_083	22	0	(22,0)	3q+1	17.2269	-7.5	0.2611	0.3610	-0.0999	Mou et al. (2026)	Fig. 5(b)	600-dpi marker digitization	Medium	No
F5V4_084	22	0	(22,0)	3q+1	17.2269	-5	0.4742	0.3610	0.1132	Mou et al. (2026)	Fig. 5(b)	600-dpi marker digitization	Medium	No
F5V4_085	22	0	(22,0)	3q+1	17.2269	-2.5	0.6383	0.3610	0.2773	Mou et al. (2026)	Fig. 5(b)	600-dpi marker digitization	Medium	No
F5V4_086	22	0	(22,0)	3q+1	17.2269	0	0.3610	0.3610	0.0000	Mou et al. (2026)	Fig. 5(b)	600-dpi marker digitization	Medium	No
F5V4_087	22	0	(22,0)	3q+1	17.2269	2.5	0.1296	0.3610	-0.2314	Mou et al. (2026)	Fig. 5(b)	600-dpi marker digitization	Medium	No
F5V4_088	22	0	(22,0)	3q+1	17.2269	5	0.1449	0.3610	-0.2161	Mou et al. (2026)	Fig. 5(b)	600-dpi marker digitization	Medium	No
F5V4_089	22	0	(22,0)	3q+1	17.2269	7.5	0.4227	0.3610	0.0617	Mou et al. (2026)	Fig. 5(b)	600-dpi marker digitization	Medium	No
F5V4_090	22	0	(22,0)	3q+1	17.2269	10	0.2426	0.3610	-0.1184	Mou et al. (2026)	Fig. 5(b)	600-dpi marker digitization	Medium	No
F5V4_136	23	0	(23,0)	3q+2	18.0100	-10	0.5180	0.4740	0.0440	Mou et al. (2026)	Fig. 5(c)	600-dpi marker digitization	Regular	No
F5V4_137	23	0	(23,0)	3q+2	18.0100	-7.5	0.2920	0.4740	-0.1820	Mou et al. (2026)	Fig. 5(c)	600-dpi marker digitization	Regular	No
F5V4_138	23	0	(23,0)	3q+2	18.0100	-5	0.0470	0.4740	-0.4270	Mou et al. (2026)	Fig. 5(c)	600-dpi marker digitization	Regular	No
F5V4_139	23	0	(23,0)	3q+2	18.0100	-2.5	0.1670	0.4740	-0.3070	Mou et al. (2026)	Fig. 5(c)	600-dpi marker digitization	Regular	No
F5V4_140	23	0	(23,0)	3q+2	18.0100	0	0.4740	0.4740	0.0000	Mou et al. (2026)	Fig. 5(c)	600-dpi marker digitization	Regular	No
F5V4_141	23	0	(23,0)	3q+2	18.0100	2.5	0.5060	0.4740	0.0320	Mou et al. (2026)	Fig. 5(c)	600-dpi marker digitization	Regular	No
F5V4_142	23	0	(23,0)	3q+2	18.0100	5	0.2040	0.4740	-0.2700	Mou et al. (2026)	Fig. 5(c)	600-dpi marker digitization	Regular	No
F5V4_143	23	0	(23,0)	3q+2	18.0100	7.5	0.0960	0.4740	-0.3780	Mou et al. (2026)	Fig. 5(c)	600-dpi marker digitization	Regular	No
F5V4_144	23	0	(23,0)	3q+2	18.0100	10	0.3150	0.4740	-0.1590	Mou et al. (2026)	Fig. 5(c)	600-dpi marker digitization	Regular	No

Supplementary Table S2. Complete target–descriptor–model comparison

Repeated-cross-validation values summarize 100 validation folds generated from 20 repetitions of shuffled five-fold cross-validation. LOCO values summarize 16 held-out chirality folds. Standardization was performed within training folds for OLS, ridge regression, and support vector regression. “Other settings default” refers to scikit-learn 1.6.1 defaults.

Target	Descriptor set	Model	Exact hyperparameters	CV mean R^2	CV SD	CV MAE (eV)	CV RMSE (eV)	LOCO mean R^2	LOCO SD	LOCO MAE (eV)	LOCO RMSE (eV)
Absolute Eg	n + strain	Mean baseline	DummyRegressor(strategy="mean")	-0.0370	0.0500	0.2328	0.2835	-0.2374	0.2512	0.2333	0.2758
Absolute Eg	n + strain	OLS	StandardScaler → LinearRegression(fit_intercept=True)	0.0616	0.1137	0.2231	0.2691	-0.1733	0.4061	0.2251	0.2647
Absolute Eg	n + strain	Ridge regression	StandardScaler → Ridge(alpha=1.0, fit_intercept=True)	0.0619	0.1124	0.2230	0.2691	-0.1719	0.4017	0.2250	0.2646
Absolute Eg	n + strain	Support vector regression	StandardScaler → SVR(kernel="rbf", C=1.0, epsilon=0.1, gamma="scale")	-0.0520	0.1950	0.2397	0.2836	-0.5152	0.6705	0.2555	0.2957
Absolute Eg	n + strain	Random forest regression	RandomForestRegressor(n_estimators=300, min_samples_leaf=3, random_state=42; other settings default)	-0.0840	0.1893	0.2458	0.2881	-0.7163	0.4475	0.2845	0.3251
Absolute Eg	n + strain	Gradient boosting regression	GradientBoostingRegressor(random_state=42; other settings default)	-0.2718	0.3246	0.2674	0.3098	-1.2103	1.1320	0.3049	0.3531
Absolute Eg	diameter + strain	Mean baseline	DummyRegressor(strategy="mean")	-0.0370	0.0500	0.2328	0.2835	-0.2374	0.2512	0.2333	0.2758
Absolute Eg	diameter + strain	OLS	StandardScaler → LinearRegression(fit_intercept=True)	0.0616	0.1137	0.2231	0.2691	-0.1733	0.4061	0.2251	0.2647
Absolute Eg	diameter + strain	Ridge regression	StandardScaler → Ridge(alpha=1.0, fit_intercept=True)	0.0619	0.1124	0.2230	0.2691	-0.1719	0.4017	0.2250	0.2646
Absolute Eg	diameter + strain	Support vector regression	StandardScaler → SVR(kernel="rbf", C=1.0, epsilon=0.1, gamma="scale")	-0.0520	0.1950	0.2397	0.2836	-0.5152	0.6705	0.2555	0.2957
Absolute Eg	diameter + strain	Random forest regression	RandomForestRegressor(n_estimators=300, min_samples_leaf=3, random_state=42; other settings default)	-0.0821	0.1896	0.2456	0.2878	-0.6709	0.4399	0.2807	0.3198
Absolute Eg	diameter + strain	Gradient boosting regression	GradientBoostingRegressor(random_state=42; other settings default)	-0.2642	0.3221	0.2669	0.3088	-1.0962	1.1392	0.2981	0.3417
Absolute Eg	family indicators + strain	Mean baseline	DummyRegressor(strategy="mean")	-0.0370	0.0500	0.2328	0.2835	-0.2374	0.2512	0.2333	0.2758
Absolute Eg	family indicators + strain	OLS	StandardScaler → LinearRegression(fit_intercept=True)	0.0288	0.1056	0.2251	0.2738	-0.2404	0.5539	0.2286	0.2707
Absolute Eg	family indicators + strain	Ridge regression	StandardScaler → Ridge(alpha=1.0, fit_intercept=True)	0.0291	0.1047	0.2250	0.2738	-0.2393	0.5510	0.2286	0.2706
Absolute Eg	family indicators + strain	Support vector regression	StandardScaler → SVR(kernel="rbf", C=1.0, epsilon=0.1, gamma="scale")	0.3701	0.1431	0.1740	0.2185	0.0092	0.8727	0.1825	0.2211
Absolute Eg	family indicators + strain	Random forest regression	RandomForestRegressor(n_estimators=300, min_samples_leaf=3, random_state=42; other settings default)	0.4519	0.1199	0.1620	0.2041	0.2582	0.8124	0.1584	0.1882
Absolute Eg	family indicators + strain	Gradient boosting regression	GradientBoostingRegressor(random_state=42; other settings default)	0.4450	0.1553	0.1579	0.2040	0.2131	0.8859	0.1585	0.1865
Absolute Eg	family indicators + strain + family×strain	Mean baseline	DummyRegressor(strategy="mean")	-0.0370	0.0500	0.2328	0.2835	-0.2374	0.2512	0.2333	0.2758
Absolute Eg	family indicators + strain + family×strain	OLS	StandardScaler → LinearRegression(fit_intercept=True)	0.0716	0.1639	0.2188	0.2667	-0.2601	0.7976	0.2211	0.2645
Absolute Eg	family indicators + strain + family×strain	Ridge regression	StandardScaler → Ridge(alpha=1.0, fit_intercept=True)	0.0723	0.1624	0.2187	0.2666	-0.2579	0.7921	0.2211	0.2644
Absolute Eg	family indicators + strain + family×strain	Support vector regression	StandardScaler → SVR(kernel="rbf", C=1.0, epsilon=0.1, gamma="scale")	0.4681	0.1318	0.1575	0.2004	0.1215	0.9403	0.1689	0.2013
Absolute Eg	family indicators + strain + family×strain	Random forest regression	RandomForestRegressor(n_estimators=300, min_samples_leaf=3, random_state=42; other settings default)	0.4677	0.1382	0.1566	0.2002	0.2321	0.8496	0.1573	0.1875
Absolute Eg	family indicators + strain + family×strain	Gradient boosting regression	GradientBoostingRegressor(random_state=42; other settings default)	0.4332	0.1614	0.1590	0.2060	0.2324	0.8633	0.1564	0.1859
Absolute Eg	diameter + family indicators + strain + family×strain	Mean baseline	DummyRegressor(strategy="mean")	-0.0370	0.0500	0.2328	0.2835	-0.2374	0.2512	0.2333	0.2758
Absolute Eg	diameter + family indicators + strain + family×strain	OLS	StandardScaler → LinearRegression(fit_intercept=True)	0.1261	0.1601	0.2142	0.2585	-0.2280	0.6828	0.2155	0.2573
Absolute Eg	diameter + family indicators + strain + family×strain	Ridge regression	StandardScaler → Ridge(alpha=1.0, fit_intercept=True)	0.1271	0.1584	0.2141	0.2584	-0.2243	0.6758	0.2155	0.2571
Absolute Eg	diameter + family indicators + strain + family×strain	Support vector regression	StandardScaler → SVR(kernel="rbf", C=1.0, epsilon=0.1, gamma="scale")	0.7509	0.0603	0.1129	0.1372	0.5725	0.4553	0.1196	0.1393
Absolute Eg	diameter + family indicators + strain + family×strain	Random forest regression	RandomForestRegressor(n_estimators=300, min_samples_leaf=3, random_state=42; other settings default)	0.5521	0.1085	0.1504	0.1841	0.3982	0.4062	0.1422	0.1747
Absolute Eg	diameter + family indicators + strain + family×strain	Gradient boosting regression	GradientBoostingRegressor(random_state=42; other settings default)	0.7066	0.0906	0.1206	0.1480	0.6250	0.3721	0.1115	0.1333
Absolute Eg	n + strain + strain² + n×strain	Mean baseline	DummyRegressor(strategy="mean")	-0.0370	0.0500	0.2328	0.2835	-0.2374	0.2512	0.2333	0.2758

Target	Descriptor set	Model	Exact hyperparameters	CV mean R^2	CV SD	CV MAE (eV)	CV RMSE (eV)	LOCO mean R^2	LOCO SD	LOCO MAE (eV)	LOCO RMSE (eV)
Absolute Eg	n + strain + strain² + n×strain	OLS	StandardScaler → LinearRegression(fit_intercept=True)	0.0345	0.1362	0.2268	0.2726	-0.2448	0.4360	0.2313	0.2724
Absolute Eg	n + strain + strain² + n×strain	Ridge regression	StandardScaler → Ridge(alpha=1.0, fit_intercept=True)	0.0383	0.1309	0.2264	0.2721	-0.2376	0.4206	0.2307	0.2717
Absolute Eg	n + strain + strain² + n×strain	Support vector regression	StandardScaler → SVR(kernel="rbf", C=1.0, epsilon=0.1, gamma="scale")	-0.1275	0.1879	0.2487	0.2939	-0.5817	0.7282	0.2583	0.2990
Absolute Eg	n + strain + strain² + n×strain	Random forest regression	RandomForestRegressor(n_estimators=300, min_samples_leaf=3, random_state=42; other settings default)	-0.1710	0.1899	0.2572	0.2995	-0.7781	0.4054	0.2896	0.3297
Absolute Eg	n + strain + strain² + n×strain	Gradient boosting regression	GradientBoostingRegressor(random_state=42; other settings default)	-0.4326	0.3009	0.2843	0.3298	-1.1271	0.9126	0.3033	0.3485
Absolute Eg	diameter + strain + strain² + family indicators + family×strain	Mean baseline	DummyRegressor(strategy="mean")	-0.0370	0.0500	0.2328	0.2835	-0.2374	0.2512	0.2333	0.2758
Absolute Eg	diameter + strain + strain² + family indicators + family×strain	OLS	StandardScaler → LinearRegression(fit_intercept=True)	0.1097	0.1614	0.2180	0.2607	-0.2716	0.7068	0.2209	0.2616
Absolute Eg	diameter + strain + strain² + family indicators + family×strain	Ridge regression	StandardScaler → Ridge(alpha=1.0, fit_intercept=True)	0.1111	0.1595	0.2178	0.2605	-0.2675	0.6991	0.2208	0.2614
Absolute Eg	diameter + strain + strain² + family indicators + family×strain	Support vector regression	StandardScaler → SVR(kernel="rbf", C=1.0, epsilon=0.1, gamma="scale")	0.7861	0.0586	0.1049	0.1269	0.6056	0.4251	0.1168	0.1335
Absolute Eg	diameter + strain + strain² + family indicators + family×strain	Random forest regression	RandomForestRegressor(n_estimators=300, min_samples_leaf=3, random_state=42; other settings default)	0.5642	0.1046	0.1482	0.1817	0.4174	0.3936	0.1399	0.1723
Absolute Eg	diameter + strain + strain² + family indicators + family×strain	Gradient boosting regression	GradientBoostingRegressor(random_state=42; other settings default)	0.7310	0.0810	0.1156	0.1419	0.6459	0.3090	0.1087	0.1295
ΔEg	n + strain	Mean baseline	DummyRegressor(strategy="mean")	-0.0387	0.0545	0.3094	0.3851	-1.5983	1.3616	0.3195	0.3807
ΔEg	n + strain	OLS	StandardScaler → LinearRegression(fit_intercept=True)	-0.0443	0.0950	0.3123	0.3855	-1.8643	1.7122	0.3321	0.3902
ΔEg	n + strain	Ridge regression	StandardScaler → Ridge(alpha=1.0, fit_intercept=True)	-0.0439	0.0942	0.3123	0.3854	-1.8614	1.7088	0.3320	0.3901
ΔEg	n + strain	Support vector regression	StandardScaler → SVR(kernel="rbf", C=1.0, epsilon=0.1, gamma="scale")	-0.2531	0.2594	0.3244	0.4201	-2.8868	3.4056	0.3545	0.4287
ΔEg	n + strain	Random forest regression	RandomForestRegressor(n_estimators=300, min_samples_leaf=3, random_state=42; other settings default)	0.0989	0.1805	0.2921	0.3566	-4.4411	3.9293	0.4494	0.5301
ΔEg	n + strain	Gradient boosting regression	GradientBoostingRegressor(random_state=42; other settings default)	0.3696	0.1736	0.2494	0.2957	-5.1920	4.4644	0.4806	0.5652
ΔEg	diameter + strain	Mean baseline	DummyRegressor(strategy="mean")	-0.0387	0.0545	0.3094	0.3851	-1.5983	1.3616	0.3195	0.3807
ΔEg	diameter + strain	OLS	StandardScaler → LinearRegression(fit_intercept=True)	-0.0443	0.0950	0.3123	0.3855	-1.8643	1.7122	0.3321	0.3902
ΔEg	diameter + strain	Ridge regression	StandardScaler → Ridge(alpha=1.0, fit_intercept=True)	-0.0439	0.0942	0.3123	0.3854	-1.8614	1.7088	0.3320	0.3901
ΔEg	diameter + strain	Support vector regression	StandardScaler → SVR(kernel="rbf", C=1.0, epsilon=0.1, gamma="scale")	-0.2531	0.2594	0.3244	0.4201	-2.8868	3.4056	0.3545	0.4287
ΔEg	diameter + strain	Random forest regression	RandomForestRegressor(n_estimators=300, min_samples_leaf=3, random_state=42; other settings default)	0.1054	0.1797	0.2911	0.3553	-4.0539	3.6666	0.4275	0.5127
ΔEg	diameter + strain	Gradient boosting regression	GradientBoostingRegressor(random_state=42; other settings default)	0.3712	0.1739	0.2489	0.2953	-4.5127	3.9391	0.4472	0.5367
ΔEg	family indicators + strain	Mean baseline	DummyRegressor(strategy="mean")	-0.0387	0.0545	0.3094	0.3851	-1.5983	1.3616	0.3195	0.3807
ΔEg	family indicators + strain	OLS	StandardScaler → LinearRegression(fit_intercept=True)	0.5058	0.0895	0.2180	0.2636	-0.1254	0.3265	0.2184	0.2579
ΔEg	family indicators + strain	Ridge regression	StandardScaler → Ridge(alpha=1.0, fit_intercept=True)	0.5061	0.0886	0.2180	0.2636	-0.1225	0.3205	0.2184	0.2578
ΔEg	family indicators + strain	Support vector regression	StandardScaler → SVR(kernel="rbf", C=1.0, epsilon=0.1, gamma="scale")	0.7083	0.1040	0.1623	0.1990	0.1977	0.6868	0.1617	0.1957
ΔEg	family indicators + strain	Random forest regression	RandomForestRegressor(n_estimators=300, min_samples_leaf=3, random_state=42; other settings default)	0.7609	0.0895	0.1318	0.1801	0.3536	0.6290	0.1271	0.1725
ΔEg	family indicators + strain	Gradient boosting regression	GradientBoostingRegressor(random_state=42; other settings default)	0.7506	0.0976	0.1263	0.1835	0.3467	0.6626	0.1225	0.1700
ΔEg	family indicators + strain + family×strain	Mean baseline	DummyRegressor(strategy="mean")	-0.0387	0.0545	0.3094	0.3851	-1.5983	1.3616	0.3195	0.3807
ΔEg	family indicators + strain + family×strain	OLS	StandardScaler → LinearRegression(fit_intercept=True)	0.5262	0.0965	0.2130	0.2573	-0.1450	0.4873	0.2117	0.2523
ΔEg	family indicators + strain + family×strain	Ridge regression	StandardScaler → Ridge(alpha=1.0, fit_intercept=True)	0.5266	0.0954	0.2129	0.2573	-0.1411	0.4796	0.2116	0.2521
ΔEg	family indicators + strain + family×strain	Support vector regression	StandardScaler → SVR(kernel="rbf", C=1.0, epsilon=0.1, gamma="scale")	0.7702	0.0807	0.1362	0.1768	0.3340	0.5697	0.1386	0.1778
ΔEg	family indicators + strain + family×strain	Random forest regression	RandomForestRegressor(n_estimators=300, min_samples_leaf=3, random_state=42; other settings default)	0.7639	0.0872	0.1293	0.1790	0.3543	0.6323	0.1255	0.1717
ΔEg	family indicators + strain + family×strain	Gradient boosting regression	GradientBoostingRegressor(random_state=42; other settings default)	0.7485	0.1006	0.1288	0.1841	0.3453	0.6637	0.1245	0.1700

Target	Descriptor set	Model	Exact hyperparameters	CV mean R^2	CV SD	CV MAE (eV)	CV RMSE (eV)	LOCO mean R^2	LOCO SD	LOCO MAE (eV)	LOCO RMSE (eV)
ΔEg	diameter + family indicators + strain + family×strain	Mean baseline	DummyRegressor(strategy="mean")	-0.0387	0.0545	0.3094	0.3851	-1.5983	1.3616	0.3195	0.3807
ΔEg	diameter + family indicators + strain + family×strain	OLS	StandardScaler → LinearRegression(fit_intercept=True)	0.5232	0.1001	0.2134	0.2580	-0.1507	0.4686	0.2128	0.2540
ΔEg	diameter + family indicators + strain + family×strain	Ridge regression	StandardScaler → Ridge(alpha=1.0, fit_intercept=True)	0.5236	0.0990	0.2133	0.2579	-0.1461	0.4622	0.2126	0.2538
ΔEg	diameter + family indicators + strain + family×strain	Support vector regression	StandardScaler → SVR(kernel="rbf", C=1.0, epsilon=0.1, gamma="scale")	0.8608	0.0400	0.1099	0.1384	0.5759	0.5028	0.1112	0.1353
ΔEg	diameter + family indicators + strain + family×strain	Random forest regression	RandomForestRegressor(n_estimators=300, min_samples_leaf=3, random_state=42; other settings default)	0.7974	0.0621	0.1283	0.1667	0.4328	0.5028	0.1217	0.1630
ΔEg	diameter + family indicators + strain + family×strain	Gradient boosting regression	GradientBoostingRegressor(random_state=42; other settings default)	0.8403	0.0514	0.1150	0.1478	0.3689	0.9586	0.1221	0.1558
ΔEg	n + strain + strain² + n×strain	Mean baseline	DummyRegressor(strategy="mean")	-0.0387	0.0545	0.3094	0.3851	-1.5983	1.3616	0.3195	0.3807
ΔEg	n + strain + strain² + n×strain	OLS	StandardScaler → LinearRegression(fit_intercept=True)	-0.0755	0.1042	0.3157	0.3912	-1.9358	1.8112	0.3328	0.3943
ΔEg	n + strain + strain² + n×strain	Ridge regression	StandardScaler → Ridge(alpha=1.0, fit_intercept=True)	-0.0712	0.1011	0.3153	0.3904	-1.9271	1.8043	0.3325	0.3938
ΔEg	n + strain + strain² + n×strain	Support vector regression	StandardScaler → SVR(kernel="rbf", C=1.0, epsilon=0.1, gamma="scale")	-0.3340	0.2656	0.3312	0.4338	-2.6646	2.7712	0.3469	0.4242
ΔEg	n + strain + strain² + n×strain	Random forest regression	RandomForestRegressor(n_estimators=300, min_samples_leaf=3, random_state=42; other settings default)	-0.1566	0.1939	0.3260	0.4044	-4.0570	3.5225	0.4296	0.5113
ΔEg	n + strain + strain² + n×strain	Gradient boosting regression	GradientBoostingRegressor(random_state=42; other settings default)	0.0744	0.2141	0.2954	0.3600	-5.0500	4.0846	0.4706	0.5642
ΔEg	diameter + strain + strain² + family indicators + family×strain	Mean baseline	DummyRegressor(strategy="mean")	-0.0387	0.0545	0.3094	0.3851	-1.5983	1.3616	0.3195	0.3807
ΔEg	diameter + strain + strain² + family indicators + family×strain	OLS	StandardScaler → LinearRegression(fit_intercept=True)	0.5151	0.0946	0.2179	0.2604	-0.1943	0.4801	0.2178	0.2582
ΔEg	diameter + strain + strain² + family indicators + family×strain	Ridge regression	StandardScaler → Ridge(alpha=1.0, fit_intercept=True)	0.5157	0.0934	0.2178	0.2603	-0.1893	0.4731	0.2175	0.2580
ΔEg	diameter + strain + strain² + family indicators + family×strain	Support vector regression	StandardScaler → SVR(kernel="rbf", C=1.0, epsilon=0.1, gamma="scale")	0.8800	0.0369	0.1015	0.1284	0.6361	0.4362	0.1053	0.1277
ΔEg	diameter + strain + strain² + family indicators + family×strain	Random forest regression	RandomForestRegressor(n_estimators=300, min_samples_leaf=3, random_state=42; other settings default)	0.8054	0.0635	0.1239	0.1631	0.4619	0.4702	0.1174	0.1602
ΔEg	diameter + strain + strain² + family indicators + family×strain	Gradient boosting regression	GradientBoostingRegressor(random_state=42; other settings default)	0.8471	0.0526	0.1105	0.1442	0.3642	1.0252	0.1169	0.1526

Supplementary Table S3. Chirality-specific leave-one-chirality-out results

Each subtable reports the metrics for one of the representative configurations in Table 4 of the main manuscript. Each held-out chirality contributes nine test records. The minimum ΔE_g support-vector-regression R^2 of -0.8786 occurred for the held-out $(9,0)$ chirality.

(a) ΔE_g; Ridge regression; family indicators + strain + family×strain

Chirality	Family	Diameter (Å)	R^2	MAE (eV)	RMSE (eV)
(8,0)	3q+2	6.2643	-0.0345	0.2015	0.2454
(9,0)	3q	7.0474	-0.9044	0.1501	0.1896
(10,0)	3q+1	7.8304	0.3267	0.2779	0.3235
(11,0)	3q+2	8.6135	-0.2547	0.3261	0.3846
(12,0)	3q	9.3965	-0.1116	0.2515	0.2869
(13,0)	3q+1	10.1796	0.3760	0.2275	0.2708
(14,0)	3q+2	10.9626	-0.0948	0.2591	0.2937
(15,0)	3q	11.7456	0.1640	0.2082	0.2410
(16,0)	3q+1	12.5287	0.1430	0.1943	0.2373
(17,0)	3q+2	13.3117	-0.0195	0.2023	0.2378
(18,0)	3q	14.0948	0.3595	0.1764	0.1969
(19,0)	3q+1	14.8778	-0.3670	0.1983	0.2280
(20,0)	3q+2	15.6608	-0.1635	0.1747	0.2188
(21,0)	3q	16.4439	0.3775	0.1562	0.1862
(22,0)	3q+1	17.2269	-1.1382	0.1999	0.2619
(23,0)	3q+2	18.0100	-0.9156	0.1819	0.2317

(b) ΔE_g; support vector regression; diameter + strain + strain² + family indicators + family×strain

Chirality	Family	Diameter (Å)	R^2	MAE (eV)	RMSE (eV)
(8,0)	3q+2	6.2643	0.4055	0.1481	0.1860
(9,0)	3q	7.0474	-0.8786	0.1635	0.1883
(10,0)	3q+1	7.8304	0.7124	0.1565	0.2114
(11,0)	3q+2	8.6135	0.6593	0.1431	0.2004
(12,0)	3q	9.3965	0.6617	0.1319	0.1582
(13,0)	3q+1	10.1796	0.8929	0.0983	0.1122
(14,0)	3q+2	10.9626	0.9376	0.0555	0.0701
(15,0)	3q	11.7456	0.8501	0.0789	0.1020
(16,0)	3q+1	12.5287	0.8186	0.0761	0.1092
(17,0)	3q+2	13.3117	0.8137	0.0820	0.1016
(18,0)	3q	14.0948	0.9155	0.0621	0.0715
(19,0)	3q+1	14.8778	0.8790	0.0627	0.0678
(20,0)	3q+2	15.6608	0.6541	0.1130	0.1193
(21,0)	3q	16.4439	0.7741	0.1037	0.1122
(22,0)	3q+1	17.2269	0.3720	0.1246	0.1419
(23,0)	3q+2	18.0100	0.7098	0.0849	0.0902

(c) Absolute E_g; support vector regression; diameter + strain + strain² + family indicators + family×strain

Chirality	Family	Diameter (Å)	R^2	MAE (eV)	RMSE (eV)
(8,0)	3q+2	6.2643	-0.2877	0.2624	0.2738
(9,0)	3q	7.0474	-0.5646	0.1480	0.1718
(10,0)	3q+1	7.8304	0.7863	0.1683	0.1823
(11,0)	3q+2	8.6135	0.6065	0.2006	0.2154
(12,0)	3q	9.3965	0.7012	0.1233	0.1487
(13,0)	3q+1	10.1796	0.8784	0.0880	0.1196
(14,0)	3q+2	10.9626	0.9417	0.0553	0.0678
(15,0)	3q	11.7456	0.8711	0.0786	0.0946
(16,0)	3q+1	12.5287	0.8384	0.0884	0.1031
(17,0)	3q+2	13.3117	0.7099	0.1009	0.1268
(18,0)	3q	14.0948	0.9172	0.0578	0.0708
(19,0)	3q+1	14.8778	0.8337	0.0765	0.0795
(20,0)	3q+2	15.6608	0.6054	0.1179	0.1274
(21,0)	3q	16.4439	0.7045	0.1098	0.1283
(22,0)	3q+1	17.2269	0.4793	0.1057	0.1292
(23,0)	3q+2	18.0100	0.6678	0.0880	0.0965

Supplementary Table S4. Chirality-specific E_{g0} uncertainty analysis

Results are shown for the ΔE_g support vector regression model using diameter, nominal strain, strain², family indicators, and family×strain interactions. The unperturbed reference and all Monte Carlo realizations used the same shuffled five-fold partition generated with split seed 42. Perturbations used random seed 123.

Condition	Uncertainty bound (eV)	Realizations	Mean R^2	SD R^2	Median R^2	2.5th–97.5th percentile R^2	Mean MAE (eV)	Mean RMSE (eV)
Unperturbed reference	0	1	0.8577	—	0.8577	—	0.1072	0.1370
Chirality-specific Eg0 perturbation ±0.005 eV	0.005	500	0.8576	0.0010	0.8576	0.8557–0.8595	0.1072	0.1370
Chirality-specific Eg0 perturbation ±0.010 eV	0.01	500	0.8571	0.0019	0.8571	0.8533–0.8608	0.1073	0.1372
Chirality-specific Eg0 perturbation ±0.020 eV	0.02	500	0.8555	0.0037	0.8555	0.8481–0.8625	0.1077	0.1379

Supplementary References

[1] Mou L, Luo X, Qiu Z, Chen Z, Zheng W, Xie Q. DFT modeling of the effect of biaxial strain on the electronic structure of zig-zag SWCNT for applications as nanoelectronic switches. ACS Applied Nano Materials. 2026;9(7):3315–3323. https://doi.org/10.1021/acsanm.5c05486.
